# Bioinspired Mechanically Robust and Recyclable Hydrogel Microfibers Based on Hydrogen‐Bond Nanoclusters

**DOI:** 10.1002/advs.202401278

**Published:** 2024-04-15

**Authors:** Jingye Liang, Jishuai Xu, Jingxuan Zheng, Lijuan Zhou, Weiping Yang, Enzhao Liu, Yutian Zhu, Qiang Zhou, Yong Liu, Run Wang, Zunfeng Liu

**Affiliations:** ^1^ School of Textile Science and Engineering Tiangong University 399 West Binshui Road Tianjin 300387 China; ^2^ Tianjin Key Laboratory of Ionic‐Molecular Function of Cardiovascular disease Department of Cardiology Tianjin Institute of Cardiology the Second Hospital of Tianjin Medical University Tianjin 300211 China; ^3^ College of Materials Chemistry and Chemical Engineering Hangzhou Normal University Hangzhou 311121 China; ^4^ Department of Orthopaedics Tianjin First Central Hospital Nankai University Tianjin China; ^5^ State Key Laboratory of Medicinal Chemical Biology Key Laboratory of Functional Polymer Materials College of Chemistry Frontiers Science Center for New Organic Matter Nankai University 94 Weijin Road Tianjin 300071 China

**Keywords:** damping, hydrogel microfibers, hydrogen‐bond nanoclusters, recyclable

## Abstract

Mechanically robust hydrogel fibers have demonstrated great potential in energy dissipation and shock‐absorbing applications. However, developing such materials that are recyclable, energy‐efficient, and environmentally friendly remains an enormous challenge. Herein, inspired by spider silk, a continuous and scalable method is introduced for spinning a polyacrylamide hydrogel microfiber with a hierarchical sheath‐core structure under ambient conditions. Applying pre‐stretch and twist in the as‐spun hydrogel microfibers results in a tensile strength of 525 MPa, a toughness of 385 MJ m^−3^, and a damping capacity of 99%, which is attributed to the reinforcement of hydrogen‐bond nanoclusters within the microfiber matrix. Moreover, it maintains both structural and mechanical stability for several days, and can be directly dissolved in water, providing a sustainable spinning dope for re‐spinning into new microfibers. This work provides a new strategy for the spinning of robust and recyclable hydrogel‐based fibrous materials.

## Graphical abstract

In this work, inspired by spider silk, a mechanically robust and green recyclable hydrogel microfiber with sheath‐core structure and hydrogen‐bond nanoclusters is presented, which maintains both structural and mechanical stability for several days. The enhanced mechanical properties can be obtained by applying the pre‐stretch and twist in microfibers, which is comparable to spider silk.

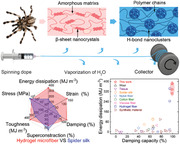

## 1 Introduction

**Figure 1:**
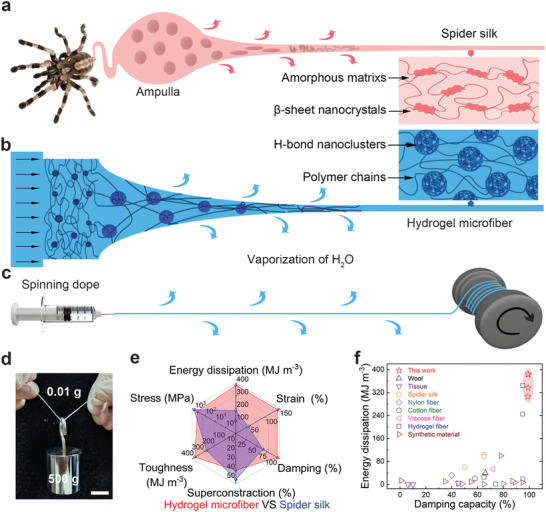
Schematic spinning of mechanically robust hydrogel microfibers inspired by spider silk. a) Schematic diagram of a spider spinning process and spider silk with a hierarchical structure consisted of β‐sheet nanocrystals and amorphous matrices. b) Schematic draw spinning and c) continuous and scalable production of hydrogel microfibers with hierarchical hydrogen‐bond nanoclusters. d) Optical image of the hydrogel yarn carrying an object over 50 000 times its weight without breaking (Scale bar = 2 cm). e) Comparison between the hydrogel microfiber and spider silk in terms of the strength, elongation, toughness, damping capacity, and supercontraction. f) Comparison of the energy‐dissipation and damping capacity of hydrogel microfibers with those of other materials in the literature.

Natural fibrous materials serve as a great source of inspiration for diverse advanced material designs, such as spider silk.^[^ ^1^ ^]^ Due to the hierarchical structure of spider silk,^[^ ^2^, ^3^, ^4^, ^5^ ^]^ including β‐sheet nanocrystal, semi‐amorphous protein matrix, and sheath‐core structure, it displays unique combinations of tensile strength, extensibility and toughness, as shown in **Figure** **1**a. Biomimicking the hierarchical structure of spider silk provides a promising strategy to construct structural fiber materials, which can be used in energy‐dissipation and shock‐absorbing applications.^[^ ^6^, ^7^, ^8^, ^9^ ^]^ However, tremendous efforts have been focused on the development of spider silk‐like fibers based on regenerated silk proteins,^[^ ^10^, ^11^, ^12^ ^]^ and thus using a non‐polypeptide approach to prepare those fibers is still difficult.

Recently, hydrogel fibers have attracted increasing attention as natural structural fiber materials, because of their high water content, high elasticity, and satisfactory mechanical properties. There are several spinning technologies for the fabrication of those fibers, such as highly oriented polyacrylonitrile hydrogel fibers, hierarchical polyvinyl alcohol hydrogel fibers, and helical sodium alginate hydrogel fibers, by electro‐spinning,^[^ ^13^, ^14^ ^]^ wet spinning,^[^ ^15^, ^16^, ^17^ ^]^ and microfluidic spinning,^[^ ^18^, ^19^, ^20^ ^]^ etc. Although the above‐mentioned fibers showcase excellent mechanical properties in terms of strength, elongation, and thus toughness, the many methods currently employed are limited by high energy consumption, complex procedures, and the use of toxic chemical solvents.

Spider spinning systems are highly energy efficient with ultralow carbon footprints,^[^ ^21^ ^]^ and spider silk naturally decomposes in the wild. A series of pioneering works on spider silk‐like fibers achieved through draw spinning have been demonstrated. Polyacrylic acid (PAA) hydrogel fibers with twisted sheath‐core structure, intra‐molecular cross‐linked nanogel fibers, buckled sheath structured polyrotaxane (PR) hydrogel fibers and supramolecular hydrogel (SPCH) fibers, exhibit excellent strength and toughness comparable to that of spider silk.^[^ ^22^, ^23^, ^24^, ^25^ ^]^ However, those are still difficult to recycle because of the introduction of many complex chemical cross‐links. Therefore, the development of mechanically robust hydrogel fibers that are recyclable, energy‐efficient, and environmentally friendly remains a challenge.

Nanoconfined structures utilizing hydrogen bonding have emerged as a novel method for preparing high‐performance polymeric materials. The hydrogen bonds (H‐bond) cross‐linking of polymeric materials is roughly realized via two major approaches: self‐association of polymers and the addition of external H‐bond cross‐linkers,^[^ ^26^ ^]^ such as nanoconfined hierarchical structured hydrogel microfibers, self‐healing nanocomposites by high‐density noncovalent bonds, dynamic H‐bond nanoconfined polyvinyl alcohol nanocomposites, and polyvinyl alcohol nanocomposites cross‐linked by β‐cyclodextrin etc.^[^ ^27^, ^28^, ^29^, ^30^ ^]^ When subjected to stretching, the rupture and reformation of H‐bonds operate at the molecular scale to dissipate fracture energy, leading to large deformation and high toughness of the matrix, while the nanoconfinement controls the strength and modulus by preserving the integrity of the network. Moreover, the feature of H‐bonds enables the resulting polymer to be recyclable underwater conditions. Hence, the manipulation of H‐bonds within nanoconfined domains through self‐assembly presents a novel approach for designing mechanically robust and recyclable hydrogel microfibers.

In this paper, inspired by the hierarchical structure and spinning process of spider silk, we present a facile, energy‐efficient draw spinning process to continuously produce high‐strength yet extensible polyacrylamide (PAM) hydrogel microfibers under ambient conditions (Figure 1b). The resulting hydrogel microfiber shows a hierarchical sheath‐core structure and H‐bond nanoclusters inside, which both are produced by the water evaporation induced self‐assembly. Through the introduction of pre‐stretch and twist to further tailor the alignment of H‐bonds, the hydrogel microfiber achieves a tensile strength of 525 MPa, a tensile strain of 138%, a toughness of 385 MJ m^−3^, and a damping capacity of 99%, which are comparable to, or even surpass those of most synthetic fibers and even spider silk (Figure 1e,f). Notably, it maintains both structural and mechanical stability for several days, and can be directly dissolved in water and re‐spinning, showing excellent recyclability.

## 2 Results and Discussion

### 2.1 Spinning Dope with Hydrogen‐Bond Nanoclusters

**Figure 2:**
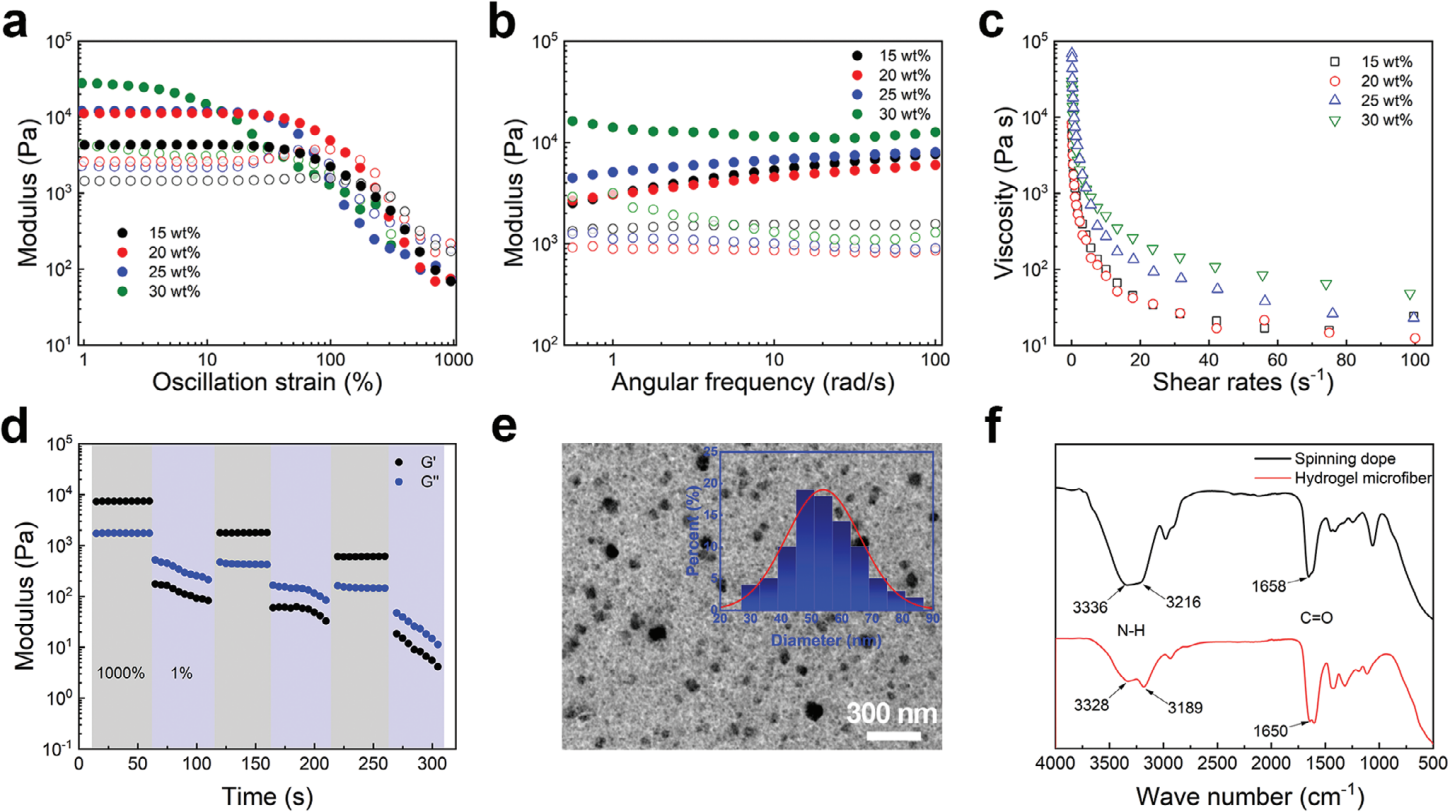
The spinnability of hydrogel spinning dopes. a) Strain oscillatory rheology and b) Frequency‐dependent rheology of spinning dopes with different monomer contents. c) Shear viscosity at 1% oscillatory strain. d) Step‐strain measurement with applied oscillatory strain between 1% and 1000% at 10 rad s^−1^ angular frequency. e) TEM image of a 20 wt.% spinning dope. f) Comparison of Fourier transform infrared spectra between the spinning dope and the hydrogel microfiber.

The spinning dope was synthesized as follows: the acrylamide monomer and initiator were added to deionized water and stirred at ambient temperature. Subsequently, the mixture was crosslinked under an ultraviolet lamp, resulting in a homogeneous and clear spinning dope (Figures S1 and S2, Supporting Information). Detailed information is available in the experimental section of the Supporting Information. To investigate spinnability, we refined the rheological behavior of the spinning dope by changing monomers content. The spinning dope could be draw‐spun to fibers when the monomer mass fraction was below 30%, as illustrated in **Figure** **2**a. The intersection of the storage modulus (G′) and loss modulus (G′′) of the spinning dope moved toward higher strains and then toward smaller strains with the increase of monomer content, which indicated that the entanglement between the polymer chains was enhanced and then weakened.^[^ ^31^ ^]^ Additionally, the spinning dope became harder as the monomer content increased, which could be seen from the increase in G′ and G′′ (Figure 2b). As shown in Figure 2c, the frequency measures illustrated the shear thinning of the spinning dope and viscosity increase, facilitating the ease of draw spinning of hydrogel microfibers.^[^ ^31^, ^32^, ^33^ ^]^ The step strain measurements between 1% and 1000% strain revealed an alternating elevation of G′ and G′′, along with a decrease in modulus, indicating the ability of the spinning dope to dissipate energy (Figure 2d). We also explored the rheological behavior of the spinning dope with different initiator content (Figures S3 and S4, Supporting Information). Transmission electron microscopy (TEM) images showed the presence of high‐density H‐bond nanoclusters, with an average radial size of 50 nm within the spinning dope with 20 wt.%, as shown in Figure 2e. Furthermore, TEM analysis showed that increasing the monomer content to 20 wt.% resulted in slight aggregation of the H‐bond nanoclusters, and high intermolecular crosslinking and large aggregates were observed when the monomer content exceeded 25 wt.% (Figure S5, Supporting Information). From the rheological test results of spinning dopes, the spinning ability of spinning dopes would be seriously affected when the size of these nanoclusters is too large. Fourier transform infrared spectra was performed to investigate the variations of the internal structure and interactions of hydrogel from spinning dope to microfibers. Upon excess water evaporation out of the hydrogel microfiber, the spontaneous nanoconfinement (i.e. H‐bond nanoclusters) of PAM chains naturally occurred. In this process, H‐bonds were formed between the C═O and ─NH_2_ on the molecular chain of acrylamide, which further aggregated the molecular chain, resulting in the formation of nanoclusters with an abundant number of H‐bonds.^[^ ^30^ ^]^ The C═O and N─H stretching vibration of the spinning dope was shifted to lower wave numbers significantly, owing to strengthened H‐bond interactions between polymer chains, as shown in Figure 2f.^[^ ^34^, ^35^ ^]^

### 2.2 Fabrication and Sheath‐Core Structure of Hydrogel Microfibers

**Figure 3:**
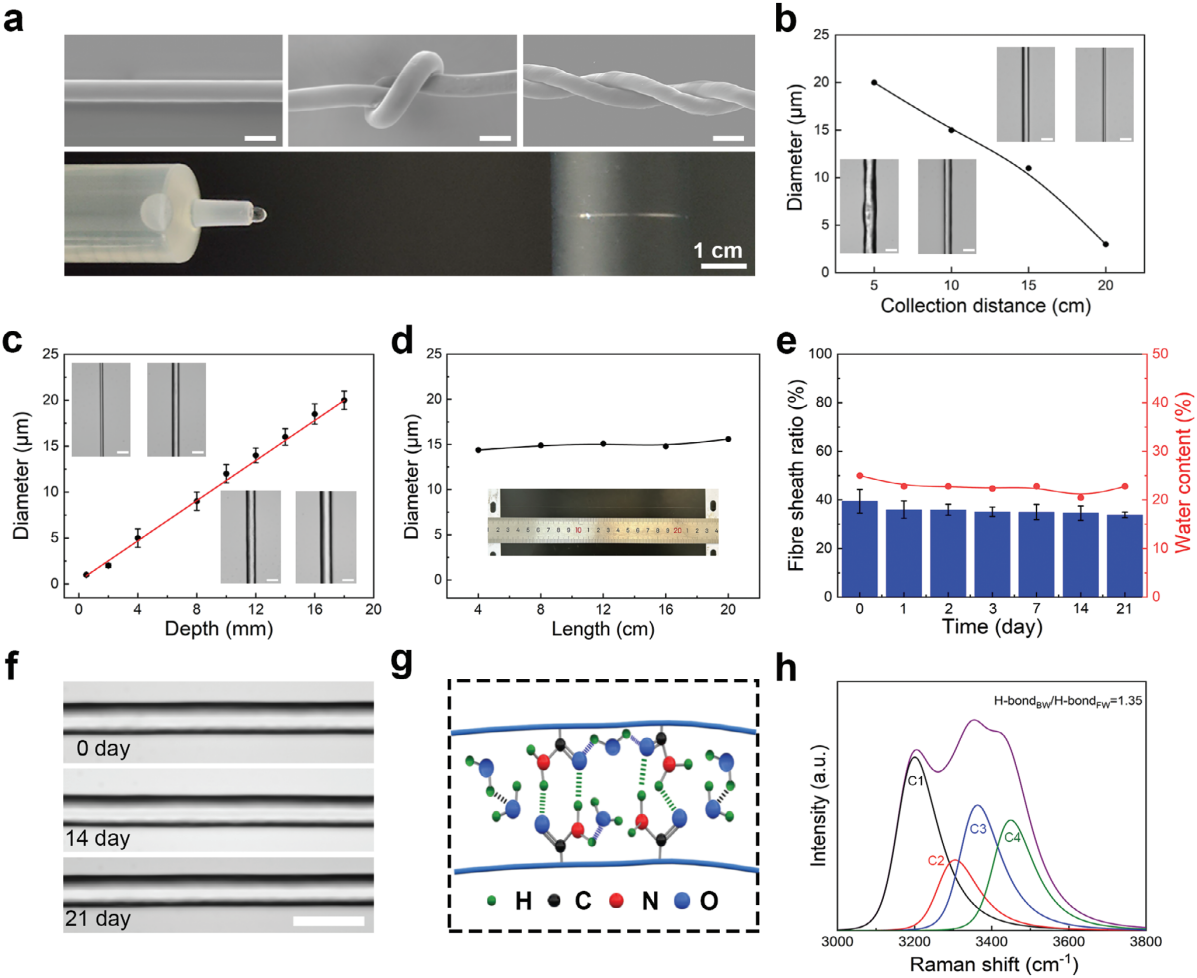
Preparation and stability of hydrogel microfibers. a) SEM images and continuous production of hydrogel microfibers. b,c) Dependency of hydrogel microfiber diameters on collection distances and the initial immersion depth of the wire, with insets showing optical microscope images of produced hydrogel microfibers (Scale bar = 20 µm). d) Diameter distribution of the hydrogel microfiber. e) Profile of the hydrogel microfiber sheath ratio and water content as function of time. f) Optical images of hydrogel microfibers in the air for 21 days (Scale bar = 20 µm). g) Schematic diagram depicting the hydrogen‐bonds in the hydrogel microfiber. h) Raman spectra at 3300–3700 cm^−1^ and the ratio of the bound water to the free water of the hydrogel microfiber.

Hydrogel microfibers were obtained through draw spinning (Figures S6 and S7, Supporting Information), and can be continuously produced under ambient conditions (**Figure** **3**a; Video S1, Supporting Information). Scanning electron microscope (SEM) images of hydrogel microfibers showed a smooth and compact morphology, which can be knotted and plied (Figure 3a; Figure S8, Supporting Information). We attempted to collect hydrogel microfibers at different collection distances. When the collection distance was less than 5 cm, the hydrogel fiber was not uniform in diameter due to insufficient drying time, whereas a distance larger than 20 cm resulted in easy breakage during the spinning process, preventing sequential collection. With the increase in the collection distance, the diameter of the collected hydrogel microfiber decreased (Figure 3b). The diameter of hydrogel microfibers from draw spinning process, tunable from 1 to 25 µm, was linearly proportional to the dipping depth of the metal wire, as illustrated in Figure 3c. We also prepared hydrogel microfibers with a length of 25 cm fixed on the frame to observe the diameter distribution. The diameter was ≈15 µm along the whole length (Figure 3d).

During the draw spinning process, water evaporated from the spinning dope and hydrogel microfiber, leading to the alignment of polymer chains and the self‐assembly of H‐bonds into nanoclusters. Upon exposure to ambient air, the shape of as‐prepared hydrogel microfiber was promptly fixed through water evaporation, forming a stable sheath on the microfiber surface. As a result, the hydrogel microfiber showed a transparent core and an opaque sheath under metallographic microscopy in a reflective mode (Figure S9, Supporting Information). We also prepared the spinning dope using acrylic acid monomer. Although polyacrylic hydrogel microfibers can be obtained by draw spinning, unlike PAM hydrogel microfibers which can be used directly after draw spinning, they require drying in air for ≈60 min before use (Figures S10 and S11, Supporting Information). Consequently, the post‐processed time for hydrogel microfibers was significantly reduced, which was surpassing that of previously reported hydrogel fibers.^[^ ^21^, ^22^, ^36^ ^]^ Due to the hydrophilic C═O, and ─NH_2_ groups in hydrogel microfibers can bind water molecules, creating bound water that has more structural bonding than free water and is not easily evaporated, which was beneficial to maintain the structural and mechanical stability.^[^ ^37^ ^]^ Therefore, the sheath ratio and water content of the hydrogel microfiber remained nearly constant at 35 and 22% for 21 days under ambient air, respectively (Figure 3e,f; Figures S12 and S13, Supporting Information). The Raman spectra band of the hydrogel microfiber was measured and decomposed into four Gaussian components,^[^ ^38^, ^39^ ^]^ corresponding to H‐bonds of free water (FWH, 3200 cm^−1^, C1), ─NH_2_ of PAM (3304 cm^−1^, C2), H‐bonds of bounded water (BWH, 3363 cm^−1^, C3 and 3448 cm^−1^, C4), as shown in Figure 3g,h and Equation S1 (Supporting Information). The ratio of bound water to free water was 1:3 calculated from Equation S2 (Supporting Information), indicating the presence of more bound water inside the hydrogel microfiber. The differential scanning calorimetry result showed the hydrogel microfiber had a lower freezing point of −36 °C than that of the spinning dope (Figure S14, Supporting Information). Regardless of its diameter, a stable sheath ratio was consistently maintained (Figure S15, Supporting Information).

### 2.3 Mechanical Strengthening of Hydrogel Microfibers

**Figure 4:**
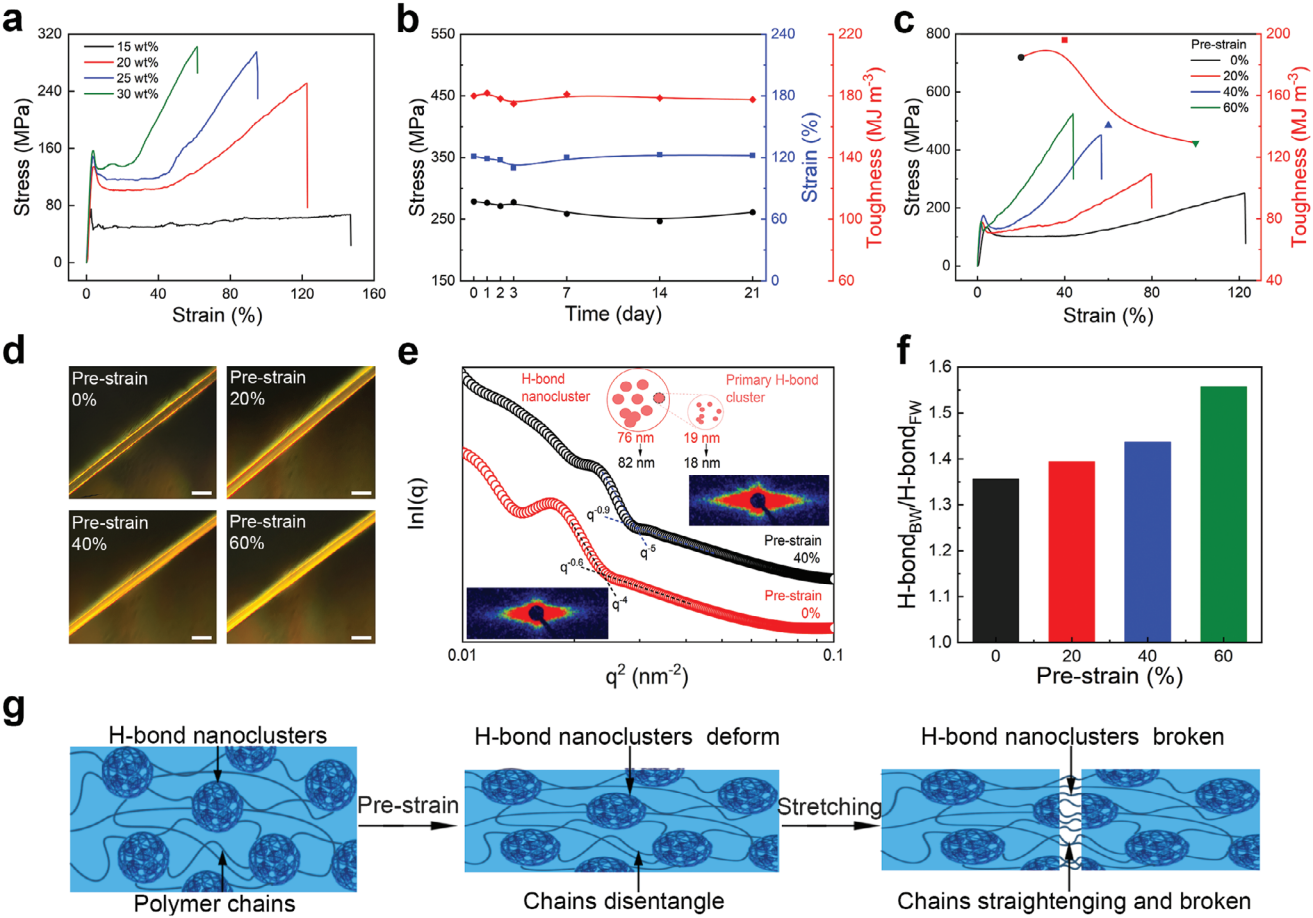
Pre‐stretching‐enhanced mechanical properties and internal interactions of hydrogel microfibers. a) Stress–strain curves of hydrogel microfibers with different monomer contents. b) Stress, strain, and toughness changes of hydrogel microfibers. c) Stress–strain curves and toughness of hydrogel microfibers with different pre‐stretch strain. d) Polarized images of hydrogel microfibers with different pre‐stretch strain (Scale bar = 20 µm). e) SXAS patterns and fitting results of hydrogel microfibers with 0and 40% pre‐stretch. f) The ratio of the bound water to the free water of hydrogel microfibers under different pre‐stretch strain. g) Schematic illustration of mechanical reinforcement and toughening mechanisms.

Unless otherwise stated, a 15 µm‐diameter and 10 mm‐length hydrogel microfiber was utilized for all the following tests (Figure S16, Supporting Information). The sheath‐core structure and H‐bond nanoclusters play a crucial role in enhancing the mechanical properties of hydrogel microfibers.^[^ ^28^ ^]^ Tensile stress–strain measurements under various monomer contents showed that the hydrogel microfiber with 20% mass fraction exhibited a tensile strength of 250 MPa, and a tensile strain of 122%, and thus the toughness of 181 MJ m^−3^ (**Figure** **4**a; Figure S17, Supporting Information). Impressively, a 500‐ply hydrogel yarn, weighing 0.01 g, successfully lifted a 500 g weight without breaking (Figure 1d). Moreover, Due to the stable sheath‐core structure, the strength, tensile, and toughness of the hydrogel microfiber remained almost unchanged for 21 days (Figure 4b; Figure S18, Supporting Information). Interestingly, the addition of various salt ions did not significantly increase the mechanical properties of hydrogel microfibers, as shown in Figures S19 and S20 (Supporting Information). After drying at 60 °C for 5 min, the tensile strength of hydrogel microfibers increased to 300 MPa. However, the tensile strain decreased to 62%, resulting in a toughness of 112 MJ m^−3^ (Figure S21, Supporting Information). The mechanical properties of hydrogel microfibers with different diameters, initiator contents, and strain rates were also investigated (Figures S22–S24, Supporting Information).

Owing to the presence of only H‐bond nanoclusters, the mechanical properties of hydrogel microfiber can be substantially altered by environmental humidity. Reducing relative humidity to below 40% caused the microfiber to be robust and tough, while increasing relative humidity led to a more extensible microfiber with decreased stress and higher elongations (Figure S25, Supporting Information). At relative humidity of 80%, the J‐shaped true stress–strain curve characteristic for strain stiffening of hydrogel microfiber indicates that even at such a high humidity (Figure S26, Supporting Information), the H‐bond nanoclusters inside the microfiber remained stable serving as strong crosslinks to restrain mechanical failure at large strains.^[^ ^40^ ^]^ Additionally, we also performed hydrophobic modification of hydrogel microfibers with hydrophobic finishing agents. These microfibers were then subjected to ambient air to investigate the morphological changes and the mechanical properties. The results showed a slight increase in strength from 261 to 276 MPa, while the tensile strain significantly decreased from 121% to 64% (Figures S27 and S28, Supporting Information).

For enhancement of the mechanical properties, manipulation of H‐bond nanoclusters and polymer chains through pre‐stretch of as‐spun hydrogel microfibers was employed.^[^ ^22^, ^23^ ^]^ A hydrogel microfiber with a pre‐stretch strain of 60% exhibited significant structural evolution, which significantly increased the fiber tensile strength to 525 MPa and decreased the fiber elongation to 43%. The maximum toughness reached 196 MJ m^−3^ with a pre‐stretch strain of 20%, as shown in Figure 4c. We also assessed the energy dissipation and the damping capacity of hydrogel microfibers. The energy dissipation of the microfibers corresponds to the energy absorbed by the microfiber during deformation from an external load. Damping capacity was evaluated as the percentage difference between loaded and unloaded energies, which is the ratio of energy dissipation to incoming energy.^[^ ^22^ ^]^ The damping capacity of the as‐spun hydrogel microfiber was maintained 90% at different loading‐unloading cycles (Figures S29 and S30, Supporting Information), which exceeded that of natural spider silk.^[^ ^24^ ^]^ The Fourier transform infrared spectra, stress–strain curves, the energy dissipation and the damping capacity of hydrogel microfibers with different pre‐stretching strains were presented in Figures S31 and S32 (Supporting Information), achieving maximum 191 MJ m^−3^ of the energy dissipation and 99% of the damping capacity at a 20% pre‐strain.

As expected, the pre‐stretch improved the orientation of the hydrogel microfiber, as shown in the 2D small angle X‐ray scattering (SAXS) pattern and the polarized images at the azimuth angles of 45° (Figure 4d,e). In the 1D SAXS pattern, q^−4^ scattering emerged in the low‐q regime, showing a dense aggregation of H‐bond nanoclusters.^[^ ^28^ ^]^ The observed peak near q = 0.13 nm^−1^ corresponds to a distance between neighboring H‐bond nanoclusters of ≈48 nm. SAXS fitting curves showed an average size of primary H‐bond clusters of 19 nm and H‐bond nanocluster aggregates of 76 nm for as‐spun hydrogel microfibers. For hydrogel microfibers with a pre‐stretch strain of 40%, the size of H‐bond nanocluster aggregates increased to 82 nm, and the distance between them decreased to 42 nm (Figure 4e; Figure S33, Supporting Information).^[^ ^27^, ^41^, ^42^, ^43^ ^]^ The toughness of hydrogel microfibers reached the highest value when the size of H‐bond nanoclusters is in the interval of 76–82 nm. Further increasing the size of the nanoclusters in the hydrogel microfiber resulted in increased strength but decreased tensile strength and toughness. The 2D Wide‐angle X‐ray Scattering indicated the potential existence of a short‐range ordered structure within the fibers due to the presence of H‐bond nanoclusters (Figure S34, Supporting Information).^[^ ^28^ ^]^ These structural changes in the self‐assemblies of H‐bonds correlated with the dependence of mechanical properties on the pre‐stretch strain. Raman spectra was employed to demonstrate the effect of pre‐stretch on the interaction between polymer chains and water in hydrogel microfibers. The characteristic peaks of the C═O, C─N, and ─CH_3_ bonds were shifted to lower wavelengths with the microfiber pre‐stretch strain increased (Figure S35, Supporting Information). This shift can be explained by the frequency of vibration and the vibrational force constant using Equation S1 (Supporting Information).^[^ ^44^ ^]^ The variation of H‐bond during microfiber pre‐stretch was determined by the ratio of Raman peak areas (Figure S36; Equation S2, Supporting Information).^[^ ^39^, ^45^, ^46^ ^]^ With the pre‐stretch strain increased, the ratio of bound water and free water increased from 1.3 to 1.6, indicating improved the orientation of hydrogel microfibers, consistent with SAXS patterns and polarized images observations, as shown in Figure 4f. The formation of H‐bonds significantly enhanced the interchain interactions and resulted in a much higher areal density of the polymer chains. Based on the classic rubber elasticity theory, increasing the effective polymer chain density leads to the increased Young's modulus.^[^ ^47^ ^]^ Therefore, these nanoconfinement phases of high polymer concentrations can effectively restrict the chain movement and slippage serving as nanofillers to enhance the mechanical strength upon mechanical stretching. Meanwhile, the nanoconfinement phase can also deform along stretching directions and induce the polymer matrix deformation accompanied by continuous rapid rupture and reformation of H‐bonds. Therefore, fracture energy can be dissipated effectively, thus resulting in great toughness (Figure 4g).

**Figure 5:**
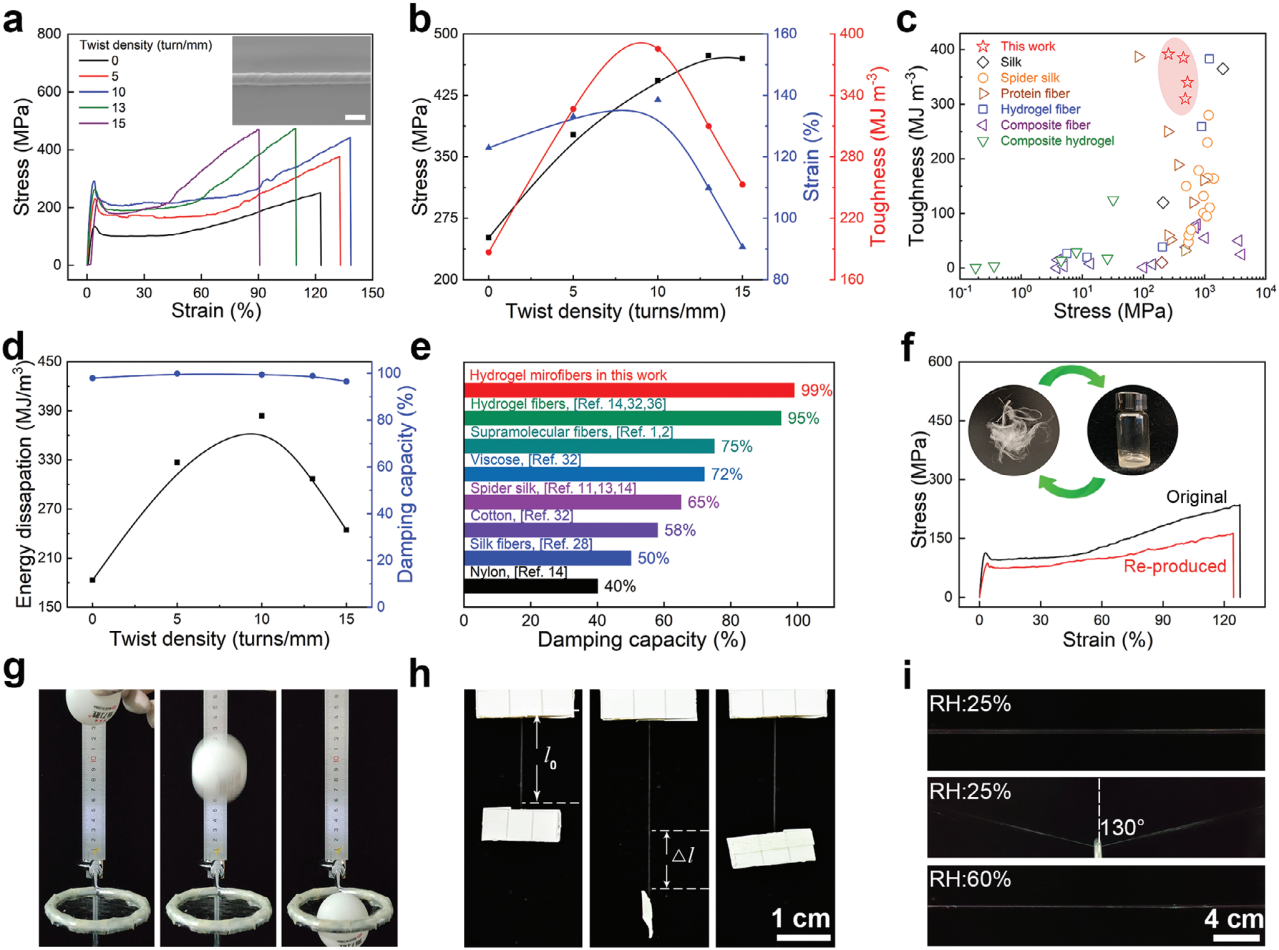
Twist‐enhanced mechanical properties and applications of hydrogel microfibers. a) SEM images and stress–strain curves of twisted hydrogel microfibers (Scale bar = 20 µm). b) Toughness of hydrogel microfibers with different twist densities. c) Comparison of the toughness and strength of hydrogel microfibers with those of other materials in the literature. d) Energy‐dissipation and damping capacity of hydrogel microfibers with different twist densities. e) Comparison of the damping capacities of hydrogel microfibers with other typical damping fiber materials. f) Stress–strain curves of the original and re‐produced hydrogel microfiber. Inset images depicting the original hydrogel microfibers and the recycled spinning dope obtained by dissolved in water. g) Impact force reduction by a net using hydrogel microfibers for a moving object under ambient condition. h,i) Moisture‐induced supercontraction and loading capacity of vertical and horizontal directions and of hydrogel microfibers.

During the spider spinning process, amino acid residues in the protein chain interacted with each other to form a helical structure.^[^ ^2^, ^48^, ^49^ ^]^ This helical structure gives the spider silk high toughness, and higher flexibility, and allows it to absorb energy and deform plastically before breaking. The SEM images showed that the as‐spun hydrogel microfiber was structurally compact yet flexible enough to be easily twisted (**Figure** **5**a; Figure S37, Supporting Information). When the twist density was up to 10 turn/mm, both breaking strength and breaking strain increased to 473 MPa and 138%, respectively, and the toughness reached 385 MJ m^−3^, representing a 2.1 times improvement compared to the as‐spun microfiber (Figure 5a,b). Further increasing the twist density led to a reduction in fracture strength and fracture strain. The insertion of twists caused the spiral alignment of H‐bond nanoclusters in the hydrogel microfiber (Figure S38, Supporting Information).^[^ ^22^, ^23^ ^]^ About the twist, the lengths of the polymer chains in the microfiber were actually elongated by forming a spiral structure during twist insertion. This may result in the following consequences at the macromolecular level. Twist‐induced elongation of the polymer chain may generate internal stress and increase the bond angle in the acrylamide chain, therefore inducing increased rigidity of the polymer chains. These twist‐induced changes at the macromolecular level may contribute to the increase in the mechanical strength.^[^ ^22^ ^]^

The tensile strength and tensile strain of such hydrogel microfibers reinforced by twist insertion are superior to those of conventional natural fibers (Table S1, Figures S39–S41, Supporting Information). The toughness of twisted hydrogel microfiber is close to the value recorded for the strongest natural spider dragline silk found to date^[^ ^50^ ^]^ and better than several synthetic fibers (Table S1, Supporting Information). In comparison to the spider silk‐like fibers produced by various methods, including poly(methylacrylic acid) (PMAA) hydrogel fibers (19.8 MJ m^−3^, 12 MPa),^[^ ^28^ ^]^ SPCH fibers (38.46 MJ m^−3^, 205 MPa),^[^ ^24^ ^]^ PAA hydrogel fibers (259 MJ m^−3^, 895 MPa),^[^ ^22^ ^]^ Nanogel fibers (383 MJ m^−3^, 1200 MPa)^[^ ^23^ ^]^ and the PR hydrogel fibers (466 MJ m^−3^, 1600 MPa),^[^ ^25^ ^]^ the hydrogel microfiber demonstrates an excellent combination of the strength and toughness (Figure 5c). Moreover, twisted hydrogel microfibers achieve the maximum energy‐dissipation of 384 MJ m^−3^ and the damping of 99% with a twist density of 10 turn/mm (Figure 5d). Such a combination of energy dissipation and damping capability is superior to that of natural spider silk and most synthetic energy‐dissipating materials,^[^ ^22^, ^23^, ^28^, ^51^, ^52^, ^53^, ^54^, ^55^, ^56^, ^57^, ^58^ ^]^ such as hydrogel (100 MJ m^−3^, 78%),^[^ ^51^ ^]^ synthetic spider silk (344.7 MJ m^−3^, 95%),^[^ ^23^ ^]^ and cushioning foam material (1.72 MJ m^−3^, 80%),^[^ ^52^ ^]^ as shown in Figure 5e and Table S2 (Supporting Information).

### 2.4 Impact Reduction Capacity, Supercontraction, and Recyclability of Hydrogel Microfibers

Spider silk, known for its incredible load‐bearing capacity, inspired the development of hydrogel microfibers that exhibit remarkable strength and versatility.^[^ ^59^ ^]^ In this work, the hydrogel yarn with 50 strands could hold 12 g objects without breaking (Figure S42 and Video S2, Supporting Information). This exceptional load‐bearing capacity proves that the hydrogel microfiber could be applied in aerospace equipment and sports protective equipment. To demonstrate the potential damping application in shock‐absorbing scenarios, a shock‐absorbing net was constructed to capture a ping‐pong ball falling from a height of 15 cm using hydrogel microfiber (Figure 5g; Video S3, Supporting Information). The ability of hydrogel microfibers to undergo heavy deformation when subjected to external forces without immediate breakage demonstrates their efficacy in absorbing and dissipating shock.

Supercontraction, a property observed in spider silk induced by humidity, is also present in hydrogel microfibers. At 60% humidity, a 40% supercontraction was also observed in our hydrogel microfiber and hydrogel yarn of vertical and horizontal directions (Figure 5h,i). This property further enhances the adaptability of hydrogel microfibers to varying environmental conditions.

In addition to their outstanding mechanical properties, the recyclability of hydrogel microfibers is a noteworthy feature. The microfibers can be dissolved in water, and the recycled spinning dope can be re‐spun into new microfibers (Figure 5f). The re‐produced microfibers still maintained good mechanical properties, with a tensile strength of 150 MPa and a tensile strain of 120%, as shown in Figure 5f. Such recyclable microfibers, coupled with exceptional mechanical and energy dissipation capabilities, are a rare achievement. The entire production and recycling process utilizes water as a green solvent, eliminating the need for additional chemicals or complex procedures. This approach provides a new strategy for environmentally friendly production of high‐performance synthetic fibers.

## 3 Conclusion

In summary, we have successfully developed a mechanically robust and environmentally friendly recyclable hydrogel microfiber with a sheath‐core structure. The manufacturing process involves a low‐cost, energy‐efficient draw spinning method conducted under ambient conditions. The incorporation of pre‐stretching and twist to tune the alignments of H‐bond nanoclusters, enables outstanding mechanical properties to the microfibers, boasting a tensile strength of 525 MPa, a tensile strain of 138%, resulting in a toughness of 385 MJ m^−3^, and an impressive damping capacity of 99%. Notably, the hydrogel microfiber exhibits exceptional recyclability, as they can be dissolved using water as a solvent and re‐spun into new fibers without significant changes in mechanical properties. This work introduces a new strategy for the spinning of robust and recyclable hydrogel‐based fibrous materials, toward in application scenarios that require resistance to impact and deformation, such as lifelines, capture nets, and parachute ropes.

## 4 Experimental Section

A detailed experimental section can be found in the Supporting Information.

## Conflict of Interest

The authors declare no conflict of interest.

## Author Contributions

R. W., Z. L., Y. L., and J. L. conceived the project. All authors contributed to the experiments, data analysis, and writing of the manuscript.

## Supporting information

Supporting Information

Supplemental Video 1

Supplemental Video 2

Supplemental Video 3

Supplemental Video 4

## Data Availability

The data that support the findings of this study are available in the supplementary material of this article.
