# Supplementary material for: Bioinspired Mechanically Robust and Recyclable Hydrogel Microfibers Based on Hydrogen‐Bond Nanoclusters
Source: Adv Sci (Weinh). 2024 Apr 15;11(23):2401278. doi: 10.1002/advs.202401278 (PMC11186113; doi:10.1002/advs.202401278)
Supplement: Supplementary file 1 — Supporting Information [file ADVS-11-2401278-s002.pdf]

ADVANCED SCIENCE
Open Access

Supporting Information

for *Adv. Sci.*, DOI 10.1002/advs.202401278

Bioinspired Mechanically Robust and Recyclable Hydrogel Microfibers Based on Hydrogen-Bond Nanoclusters

Jingye Liang, Jishuai Xu, Jingxuan Zheng, Lijuan Zhou, Weiping Yang, Enzhao Liu, Yutian Zhu, Qiang Zhou, Yong Liu, Run Wang and Zunfeng Liu*

Supporting Information

Bioinspired Mechanically Robust and Recyclable Hydrogel Microfibers Based on Hydrogen-Bond Nanoclusters

JingYe Liang[1], JiShuai Xu[1], JingXuan Zheng[1], LiJuan Zhou[1], WeiPing Yang[1], Enzhao Liu[2], Yutian Zhu[3], Qiang Zhou[4], Yong Liu[1], Run Wang[1,], and Zunfeng Liu[5]*

[1]School of Textile Science and Engineering, Tiangong University, 399 West Binshui Road, Tianjin 300387, China.

[2]Tianjin Key Laboratory of Ionic-Molecular Function of Cardiovascular disease, Department of Cardiology, Tianjin Institute of Cardiology, the Second Hospital of Tianjin Medical University, Tianjin, 300211, China.

[3]College of Materials, Chemistry and Chemical Engineering, Hangzhou Normal University, Hangzhou, 311121, China.

[4]Department of Orthopaedics, Tianjin First Central Hospital, Nankai University, Tianjin, China.

[5]State Key Laboratory of Medicinal Chemical Biology, Key Laboratory of Functional Polymer Materials, College of Chemistry Frontiers Science Center for New Organic Matter, Nankai University, 94 Weijin Road, Tianjin 300071, China.

Email: wangrun@tiangong.edu.cn

This PDF file includes:

Experimental Section (Pages S2-S3)

Characterization (Pages S3-S9)

Supplementary Equations S1 to S15 (Pages S4-S9)

Supplementary Figures S1 to S42 (Pages S10-S24)

Supplementary Tables S1 and S2 (Pages S25-26)

Supplementary Videos S1 to S4 (Pages S27)

Supplementary Reference (Pages S28-S30)

Experimental Section

Materials. 2-Hydroxy-4'-(2-hydroxyethoxy)-2-methylpropiophenone (I2959, Mv: 224.25, ≥98%, Aladdin), Acrylamide (Am, Mv: 71.08, ≥99%, Aladdin), Acrylic acid (AA, Mv: 72.06, ≥99%, Aladdin), Hydrophobic finishing agents (Hydrophobic SiO_2, DaShu). All materials as follows were purchased from commercial suppliers and used without further purification.

Preparation of Hydrogel Spinning Dopes. To prepare the spinning dope for polyacrylamide (PAM) hydrogel microfibers, 2 g Am (0.028 mol), 0.015 g I2959 (0.066×10^{-2} mol, 0.2 mol% relative to the monomer), and 8 mL (0.44 mol) water were mixed. The mixture underwent stirring at room temperature for 30 minutes, followed by a vacuum degassing process lasting 15 minutes., Subsequently, the concoction was crosslinked under an ultraviolet (UV) light (365 nm, 20 W) for 2 hours.

The preparation of spinning dopes involved careful adjustments to the monomer and initiator contents. Mass fractions of 15 wt%, 20 wt%, 25 wt%, and 30 wt% were considered for the monomer. Once the mass fractions were determined by the mechanical properties and rheological behavior, the initiator content was fine-tuned by adding initiators relative to the monomer. Specifically, initiator contents of 0.05 wt%, 0.25 wt%, 0.75 wt%, and 1.5 wt% were employed to identify the optimal properties of hydrogel microfibers achievable from spinning dopes.

To prepare the spinning dope for polyacrylic acid (PAA) hydrogel microfibers, 2 g AA (0.028 mol), 0.015 g I2959 (0.066×10^{-2} mol, 0.2 mol% relative to the monomer),

and 8 mL (0.44 mol) water were mixed. The mixture underwent stirring at room temperature for 30 minutes, followed by a vacuum degassing process lasting 15 min., Subsequently, the concoction was crosslinked under an UV light (365 nm, 20 W) for 2 hours.

Fabrication of Hydrogel Microfibers. To generate PAM and PAA hydrogel microfibers, a metal rod of 0.65 mm diameter was vertically immersed into the spinning dope and draw spinning at a constant rate. The ends of the resulting microfibers were fixed to a template to facilitate subsequent testing and processing.

Unless otherwise stated, all hydrogel microfibers with 15 μm diameter and 10 mm length were prepared from PAM spinning dopes, and all the experiments were conducted under ambient conditions, with a relative humidity of 25% and a room temperature of 20°C.

Hydrophobic Treatment of Hydrogel Microfibers. The hydrophobic silica nanocoating materials were used to coat hydrogel microfibers by spray-coating. These microfibers were then subjected to ambient air to investigate the morphological changes and the mechanical properties.

Recyclability of Hydrogel Microfibers. First, 0.052 g hydrogel microfibers were prepared by the sequential production device, and then mixed with 100 μL water to obtain a new spinning dope. The content of recycled spinning dope was 20 wt% based on the optimal mechanical properties of hydrogel microfibers. A metal rod of 0.65 mm diameter was vertically immersed into the spinning dope and draw spinning at a constant rate (10 mm s^{-1}), which produced hydrogel microfibers again.

Characterization

Rheological Measurements. Rheological measurements were conducted on an Anton Paar MCR302 rheometer. In amplitude scan mode, the oscillatory strain range was 1-1000% with an angular frequency of 10 rad s^{-1}. In frequency scan mode, the angular frequency range was 0.1-100 rad s^{-1} with an oscillatory strain of 1%. The shear rate range was 0-100 s^{-1} with an angular frequency of 10 rad s^{-1} and an oscillatory strain

of 1%. Leap strain was measured with applied oscillatory strain alternating between 1-1000% for 50 s (ω=10 rad s^{-1}).

Transmission Electron Microscopy Observation of Hydrogel Spinning Dope. A Hitachi H7650 TEM electron microscope was used for transmission electron microscopy imaging of the spinning dopes at an accelerating voltage of 100 kV. The TEM samples were obtained by UV cross-linking the copper mesh after it was fished out by immersing it in the precursor solution. The samples underwent testing directly without any other treatment.

Contact Angles Measurements. Contact angles were tested on a contact angle goniometer (KRUSS, DSA30S). The samples were prepared by constructing a hydrogel microfiber net using hydrogel microfibers, with and without hydrophobic finishing agents.

Optical images. Optical and polarized images of hydrogel microfibers were characterized using the reflection mode of a metallurgical microscope (RuiHoge HJ2). Photos and videos were obtained using iPhone and camera (Nikon 5300 D).

Scanning Electron Microscope Observation of Hydrogel Microfibers. The morphology and microstructure of as-spun hydrogel microfibers were determined using a scanning electron microscope (Flexsem1000, Regulus 8100).

Infrared Spectroscopy. Infrared spectra were performed on a Nicolet iS50 FTIR spectrometer with an attenuated total reflection (ATR) mode in the range of 400-4000 cm^{-1}.

Differential Scanning Calorimetry. Thermal behavior of the spinning dope and the hydrogel microfiber was characterized by differential scanning calorimetry (DSC, NETZSCH, DSC200F3). A 10.0 mg sample, loaded in a hermetically sealed 40 μL aluminum pan was cool from 25°C to -50°C at 5 K min^{-1}. A constant nitrogen flow rate of 50 mL min^{-1} was maintained during all runs.

Raman spectroscopy. Raman images were obtained using Raman spectroscopy (WITec aipha300). The effect of microfibers orientation on internal interactions was calculated from the Raman spectra of the C=O vibrational peaks. The relationship

between the vibrational frequency and the vibrational force constant (κ) is as follows in Equation (1):

$$v = \frac{1}{2\pi c}\sqrt{\frac{\kappa}{\mu}} \tag{S1}$$

where c represents the velocity of light and μ represents the reduced mass of diatomic molecules. The decrease in frequency indicates the decrease of the vibrational force constant, which is only related to the electron cloud density. When the electron cloud density moves between two atoms, causing an increase in the overlap of the electron cloud between the atoms, the vibrational force constant increases, and vice versa.[1-3] The original electron cloud is biased to the side of the O atom with the larger atomic mass. After the O and H atoms form a hydrogen bond, the electron cloud moves slightly away from the C atom and further away from the centers of the two atoms. Therefore, the vibrational force constant decreases. According to the relationship between the vibrational force constant and the vibrational frequency, the vibrational frequency of the C=O bond should be reduced, which is consistent with the experimental observation. The increased fiber orientation strengthens the hydrogen bonding (H-bond) interactions between the molecular chains, resulting in extended C=O bonds and lower vibrational frequencies.[4-5]

We also analyzed the Raman spectra of the samples in the range of 3800-3000 cm^{-1}. The peak fitting software (Peak fitting) was used to divide the characteristic peaks. The variation of H-bond during microfiber pre-stretch was determined by the ratio of Raman peak areas. The Equation (2) is as follows:

$$(I_{C3} + I_{C4})/I_{C1} \tag{S2}$$

Where I_{C1} represents the H-bonds of free water and I_{C3}+I_{C4} represents the H-bonds of bounded water.

Two-dimensional Wide-angle X-ray Scattering and Small-angle X-ray Scattering Measurement. Wide-angle X-ray scattering (WAXS) and small-angle X-ray scattering (SAXS) were performed on the Xeuss 2.0 system (Xenocs SA, France) equipped with a two-dimensional (2D) detector. For the WAXS, the distance from the

sample to the detector was 158 mm. For SAXS, the distance from sample to detector was 6200 mm. Image acquisition time for SAXS and WAXS was 600 s.

Tensile test. All tensile tests were performed on an electronic universal material testing machine (HY-0580) with a calibrated 5 N load cell. The microfibers for the mechanical tests were fixed with adhesive tape to a template with a gauge length of 10 mm (Figure S16). Unspecified, a 15-μm-diameter hydrogel microfiber drawn from a spinning dope with a mass fraction of 20 wt% was used for all tests with a strain rate of 0.02 s^{-1}.

Pre-stretch and Twist Insertion. A hydrogel microfiber was taped to a paper frame with a gauge length of 10 mm. Hydrogel microfibers with different pre-stretch strain were conducted by stretching on a mechanical tester. Twisted hydrogel microfibers were conducted on hydrogel microfiber with a length of 20 mm. One end of the microfiber was attached to a servomotor and the other end was loaded with a 0.25 g load. An iron stick was inserted into the load to prevent the microfiber from rotating during the twisting process (Figure S37).

Water Content Measurement. The water content of hydrogel microfibers was measured by the weight difference between the equilibrium state (m_0) and the completely dry state (m_1) of the sample at a certain humidity. Hydrogel yarn which consists of 500-ply, 15-μm-diameter, and 15-cm-long hydrogel microfibers were put under ambient conditions. These fiber samples were dried in a vacuum oven at 80°C for 12 h to remove water. The water content was calculated as $(m_0\text{-}m_1)/m_0\times100\%$ and was measured using five parallel samples. The same method was used to measure the water content of spinning dopes.

Continuous and Scalable Production of Hydrogel Microfibers. The continuous and scalable production and collection device was shown in Figure 3a and Video S1. Spinning dopes were obtained with the monomer with a mass fraction of 15 wt% (0.75 wt% initiator relative to the monomer), loaded into a 5 mL syringe (Nanjing Ximai Nanotech, XMSP-B/C), and crosslinked for 2 h under 20 W UV light. When the syringe pump was started, the microfiber was manually pulled out from the syringe tip first and

pulled onto the collection roller. The spinning process was carried out under ambient conditions. Finally, the spinning dope was injected at 50 μL min^{-1} and a 32 mm diameter collector was rotated at 15 rpm with a collection distance of 10 cm.

Calculation of the Size and Distance of H-bond Nanoclusters within the Hydrogel Microfiber from 2D SAXS Analysis.[6-8]

Guinier law is given by:

$$I(q) = I_e n^2 \exp\left(-\frac{q^2 R_g^2}{3}\right) \tag{S3}$$

where $I(q)$, I_e, n, q are the scattering intensity, the x-ray scattering intensity of a single electron, the sum of electrons in a single particle, and the scattering vector (q = 4πsinθ/λ).

Taking logarithms of Equation S3:

$$\ln\ I(q) = K - \frac{R_g^2}{3} q^2 \tag{S4}$$

Where constant K is equal to $I_e n^2$. R_g can be obtained from the following Equation(S5):

$$R_g = \sqrt{-3k} \tag{S5}$$

where k is the slope of the In$I(q)$- q^2 curve.

In the case of monodisperse distribution of the ball, the following relationship exists between the Guinier radius and the radius of the sphere, as shown in Equation (6):

$$R_g = \sqrt{3/5} R_{\text{sphere}} \tag{S6}$$

The distance between two neighboring nanoscale domains (R_d) can be roughly calculated by the following Equation (7):

$$R_d = 2\pi / q_{max} \tag{S7}$$

where q_{max} is the scattering vector at which $I_q{}^2$ shows a maximum value.

Calculations of Tensile Stress, Tensile Strain, Toughness, Energy Dissipation, Damping Capacity, and Supercontraction.[9-10]

The tensile stress (σ) of the as-prepared hydrogel microfiber was calculated by Equation (8):

$$\sigma = \frac{F}{\pi r^2} \tag{S8}$$

where F is the load force recorded by the universal machine and r is the initial radius of the generated hydrogel microfiber. The initial radius was measured at three locations under the metallurgical microscope and the average value of r.

The tensile strain (ε) of the hydrogel microfiber is calculated by Equation (9):

$$\varepsilon = \frac{l - l_0}{l_0} \times 100\% \tag{S9}$$

where l_0 is the initial length of the hydrogel microfiber between the fixtures and l is the length of the microfiber before breakage. The initial lengths of the generated microfiber were set to 10 mm for all the tensile tests.

The true stress (σ_{Ture}) was calculated by Equation (10):

$$\sigma_{\text{Ture}} = \sigma \times (1 + \varepsilon) \tag{S10}$$

The true strain ($\varepsilon_{\text{Ture}}$) was calculated by Equation (11):

$$\varepsilon_{\text{Ture}} = \ln(1 + \varepsilon) \tag{S11}$$

The toughness (E) is an engineering value used to evaluate the ability of a material to absorb energy and plastic deformation without fracture. The (E) of the generated hydrogel microfiber is calculated by Equation (12):

$$E = \int_0^{\varepsilon} \sigma d\varepsilon \tag{S12}$$

The energy-dissipation (ΔE) of the hydrogel microfiber is calculated by the area under the hysteresis curve of the stress-strain curve during loading and unloading, Equation (13):

$$\Delta E = \int_{\text{loading}} \sigma d\varepsilon - \int_{\text{unloading}} \sigma d\varepsilon \tag{S13}$$

The damping capacity (η) of the hydrogel microfiber during loading-unloading is calculated employing Equation (14):

$$\eta = \Delta E / E \times 100\% \tag{S14}$$

The supercontraction of hydrogel fibers was recorded by iPhone while being exposed to water vapor generated by a humidifier. Unless otherwise indicated, supercontraction was achieved by stretching the hydrogel fibers for a certain length after exposing them to water vapor and stopped by removing the vapor.

The supercontraction (S) was calculated using Equation (15):

$$S(\%) = (l_1 - l_2)/l_1/100\% \qquad \text{(S15)}$$

where l_1, l_2 are the lengths of the hydrogel microfiber before and during supercontraction, respectively.

Supplementary Figures

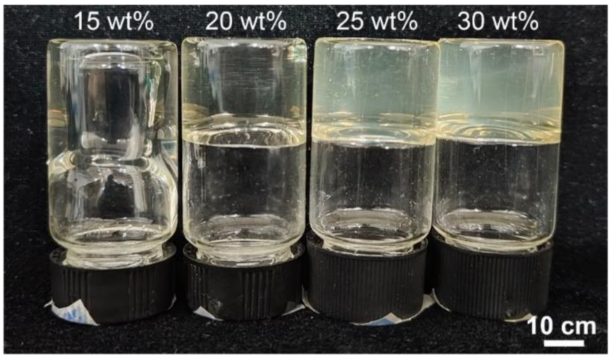

Figure S1. Photographs of spinning dopes with different monomer contents.

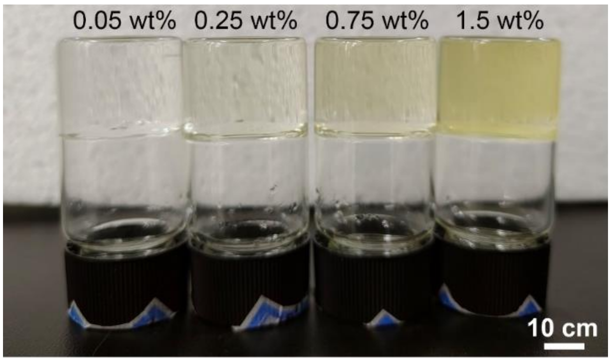

Figure S2. Photographs of spinning dopes with different initiator contents.

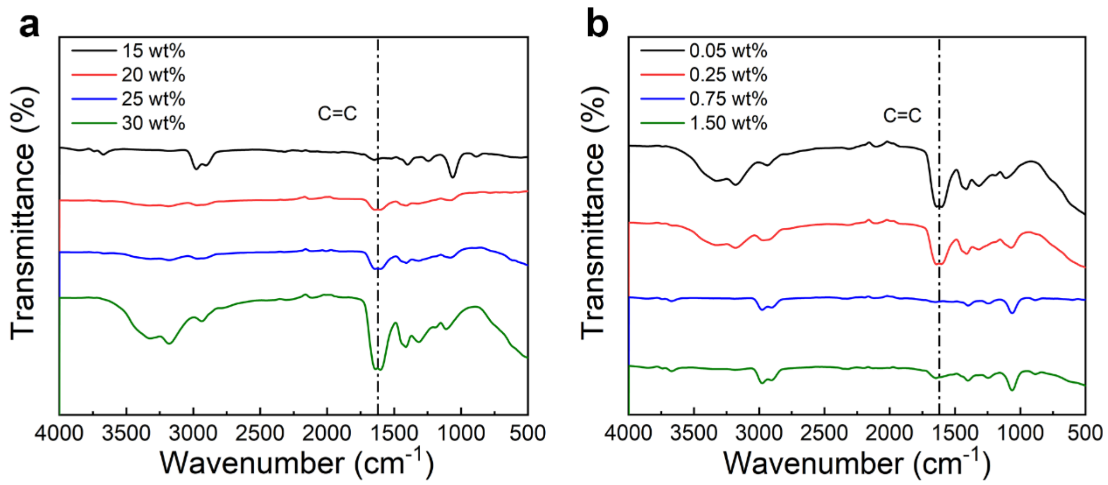

Figure S3. a) Fourier transform infrared spectra of spinning dopes with different monomer mass fractions and b) initiator contents.

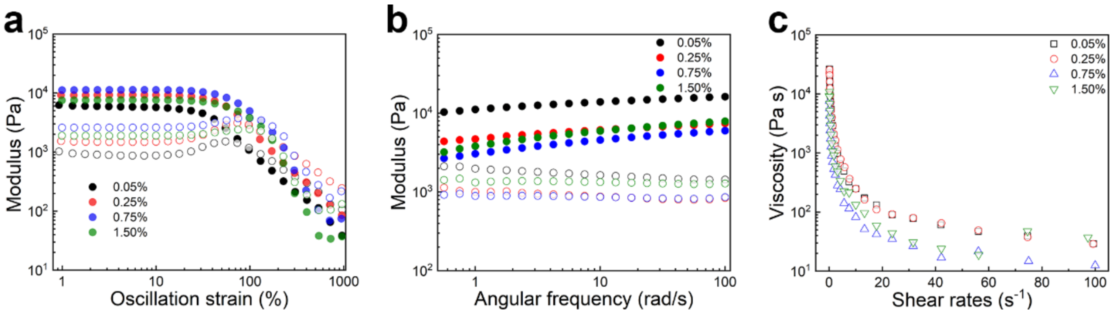

Figure S4. a) Strain oscillatory rheology, b) Frequency dependent rheology and c) Shear viscosity at 1% oscillatory strain of spinning dopes with different initiator contents.

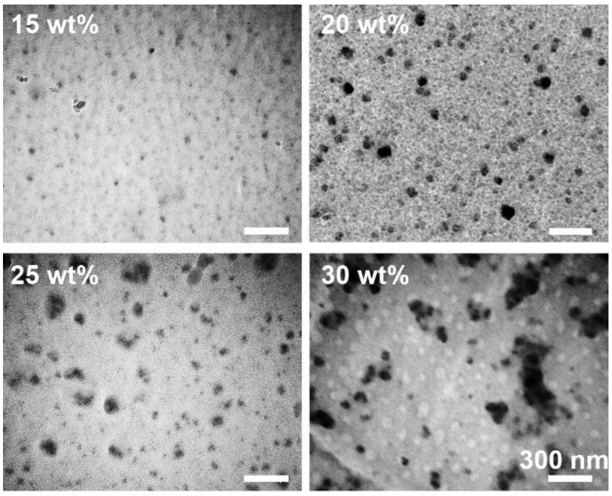

Figure S5. TEM images of spinning dopes with different monomer contents.

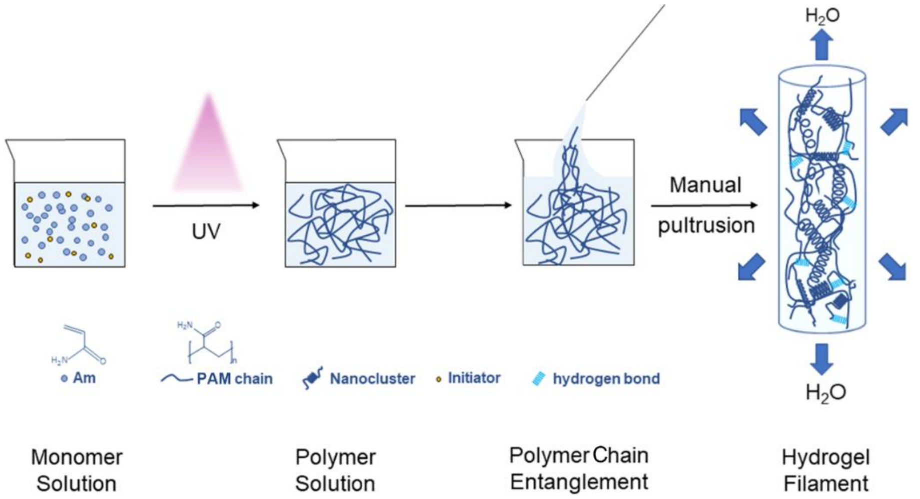

Figure S6. The synthesis and draw spinning of the spinning dope.

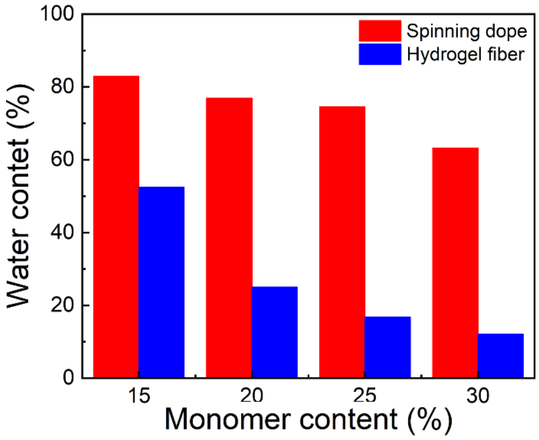

Figure S7. The water content of spinning dopes and hydrogel microfibers with different monomer contents.

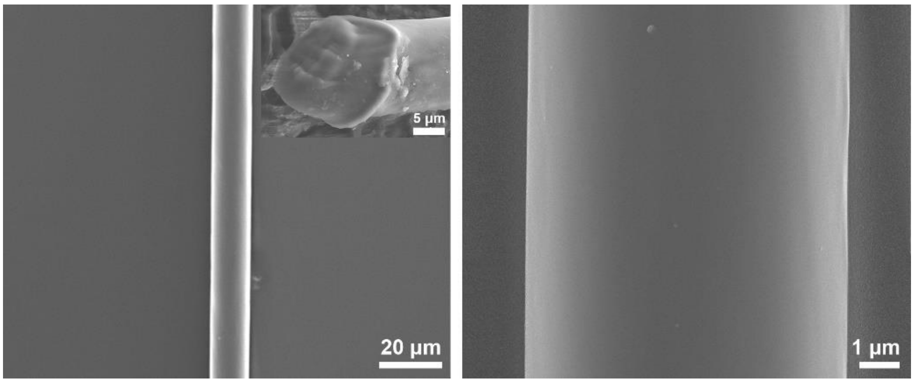

Figure S8. SEM images of surface and cross-section of hydrogel microfibers.

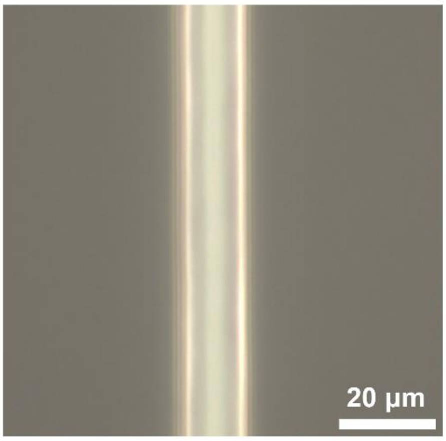

Figure S9. Raman image of the hydrogel microfiber.

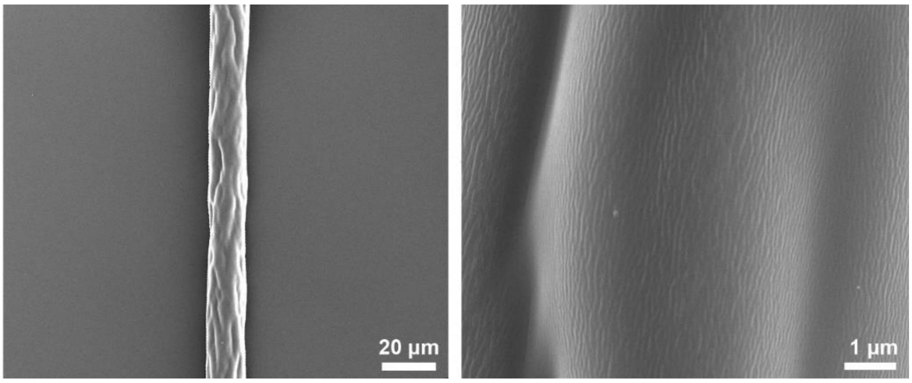

Figure S10. SEM images of the PAA hydrogel microfiber.

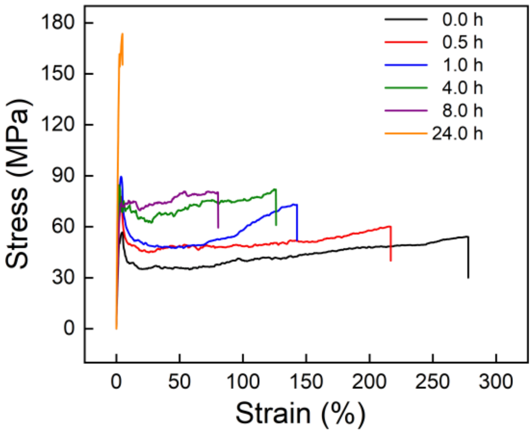

Figure S11. Stress-strain curves of PAA hydrogel microfibers with different dry time.

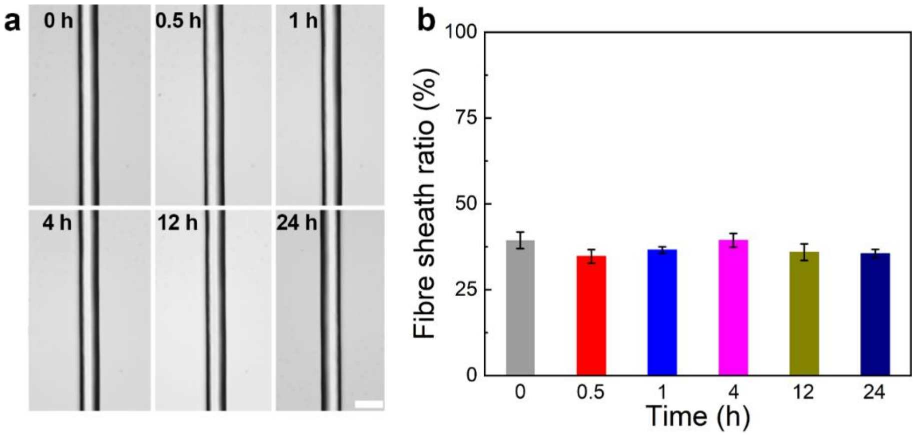

Figure S12. a) Optical images of the hydrogel microfiber after being placed in the air for 24 h (Scale bar = 20 μm), b) Profile of the hydrogel microfiber sheath ratio as function of time.

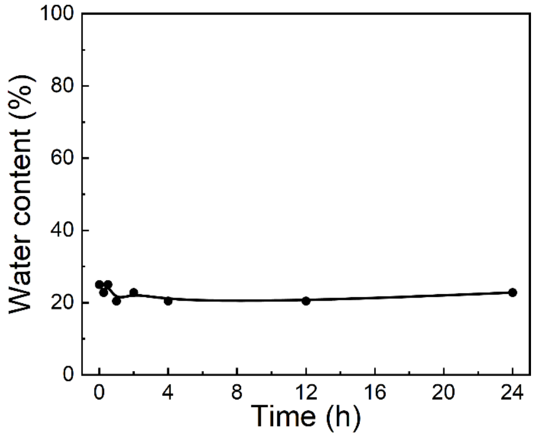

Figure S13. Changes in the water content of hydrogel microfibers under ambient conditions as function of time.

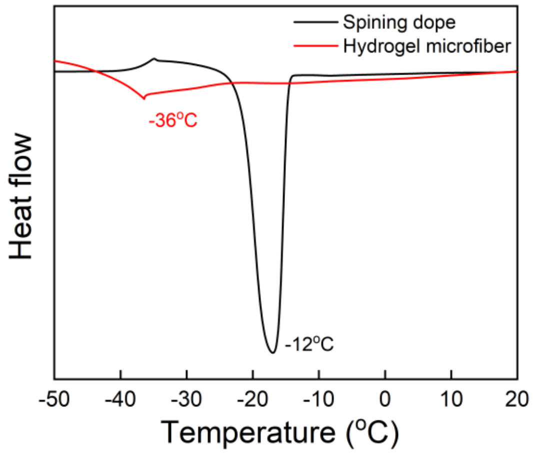

Figure S14. Differential scanning calorimetry curves of the spinning dope and the hydrogel microfiber.

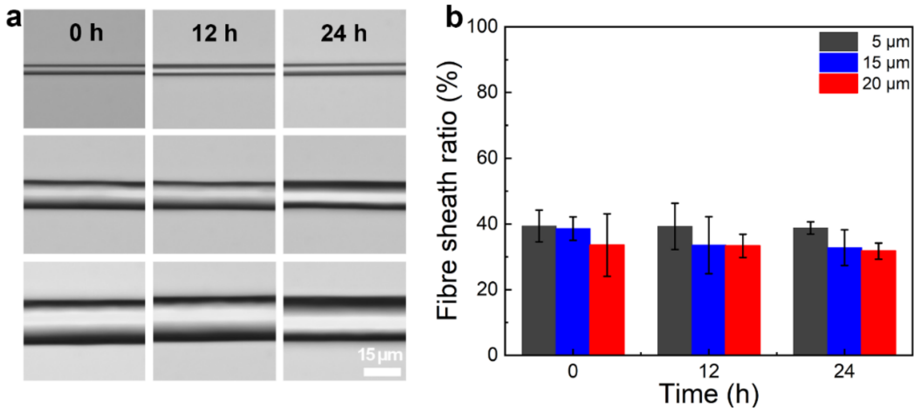

Figure S15 a) Optical images of hydrogel microfibers after being placed in the air for 24 h (Scale bar = 20 μm), b) Profile of hydrogel microfibers sheath ratio as function of time.

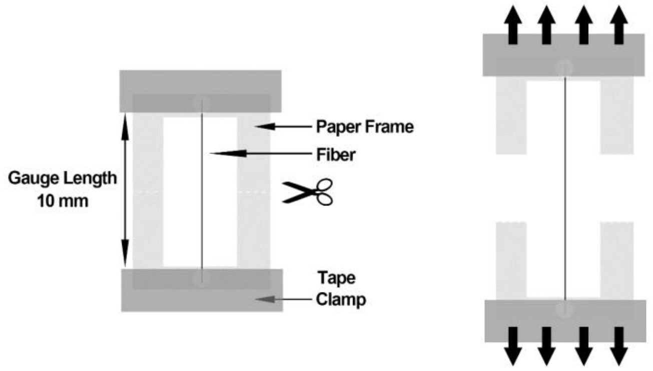

Figure S16. Schematic diagram of the hydrogel microfiber template used for mechanical testing.

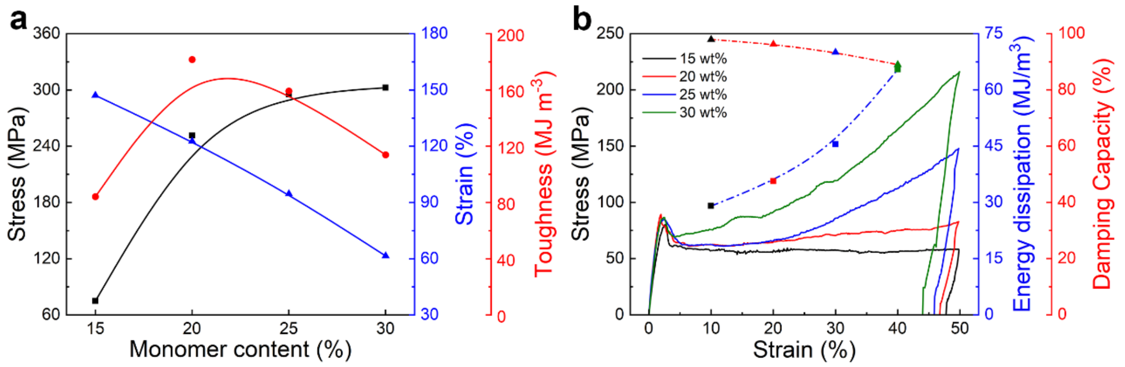

Figure S17. a) Tensile strength, tensile strain, and toughness of hydrogel microfibers with different monomer contents. b) Loading-unloading curves, energy-dissipation, and damping capacities at 50% strain of hydrogel microfibers with different monomer contents.

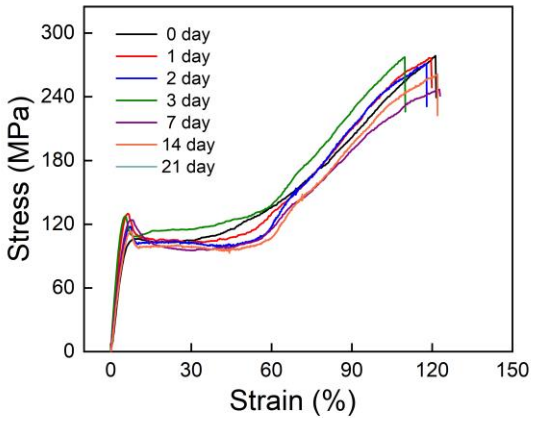

Figure S18. Stress-strain curves of hydrogel microfibers after being placed in the air for 21 days.

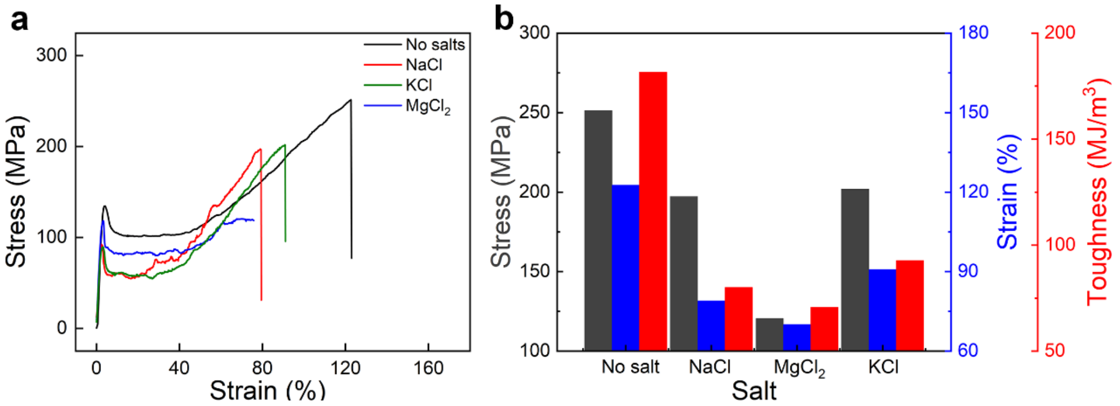

Figure S19. a) Stress-strain curves and b) Tensile stress, tensile strain, and toughness of hydrogel microfibers with different types of salts (0.1 M).

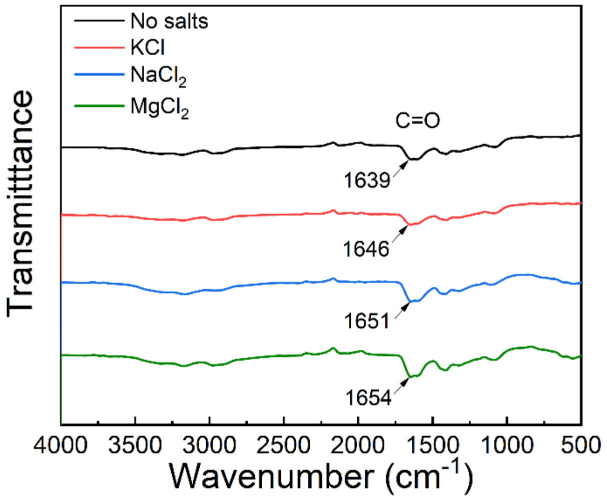

Figure S20. Fourier transform infrared spectra of spinning dopes with different types of salts.

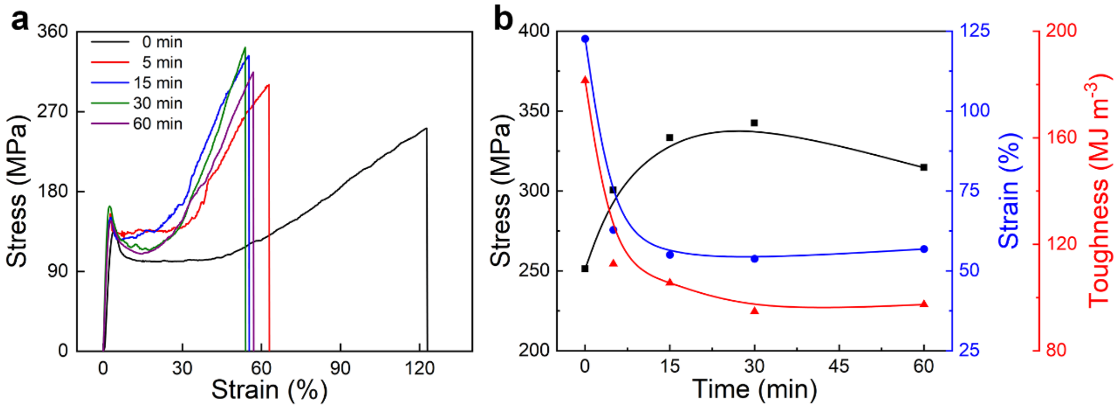

Figure S21. a) Stress-strain curves and b) Tensile stress, tensile strain, and toughness of hydrogel microfibers dried at 60°C for different time.

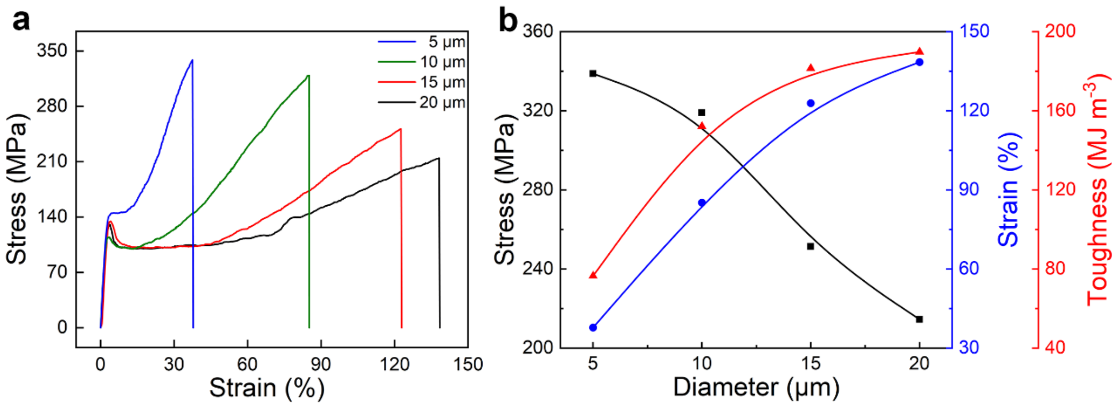

Figure S22. a) Stress-strain curves and b) Tensile stress, tensile strain, and toughness of hydrogel microfibers with different diameters.

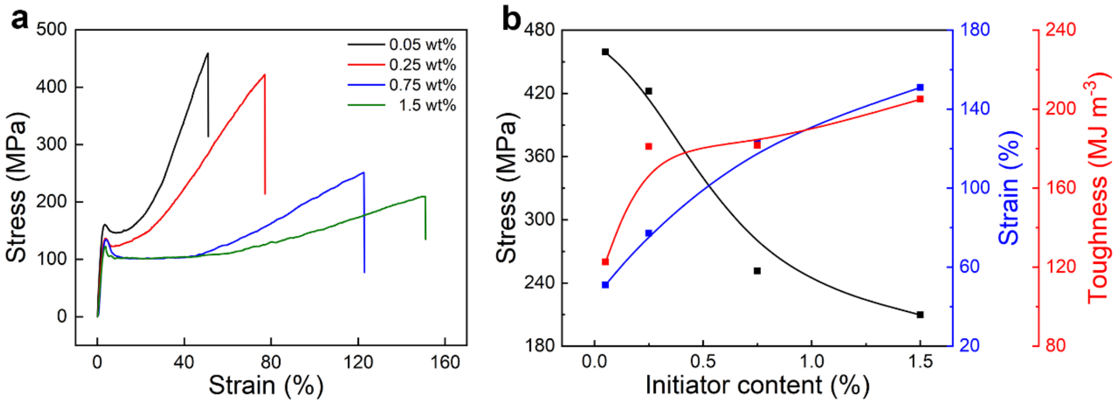

Figure S23. a) Stress-strain curves and b) Tensile stress, tensile strain, and toughness of hydrogel microfibers with different initiator contents.

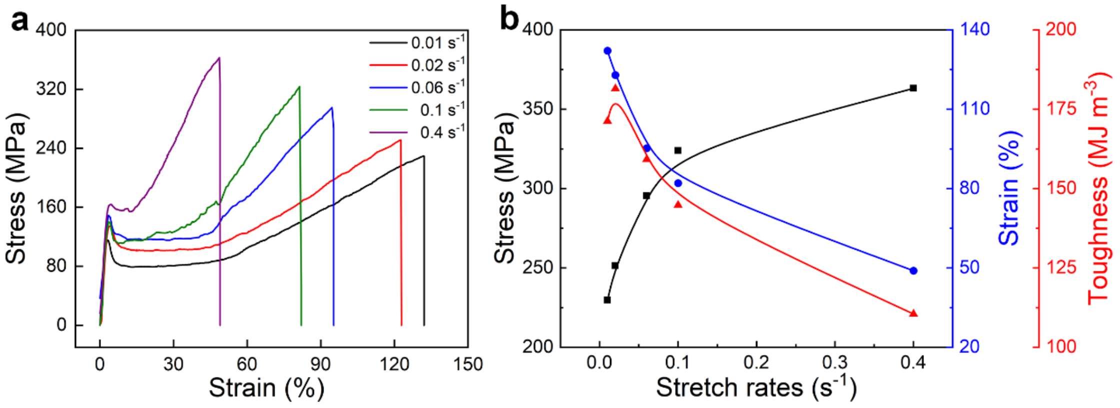

Figure S24. a) Stress-strain curves and b) Tensile strength, tensile strain, and toughness of hydrogel microfibers with different strain rates.

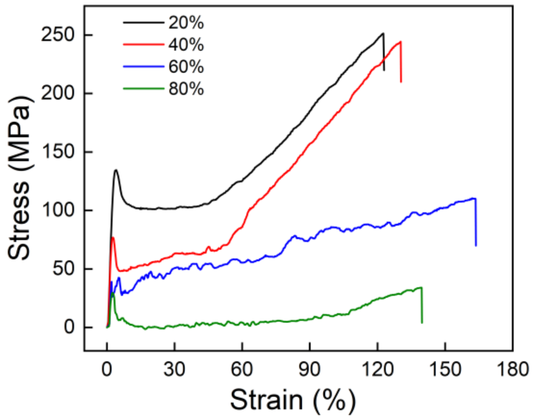

Figure S25. Stress-strain curves of hydrogel microfibers at different humidity.

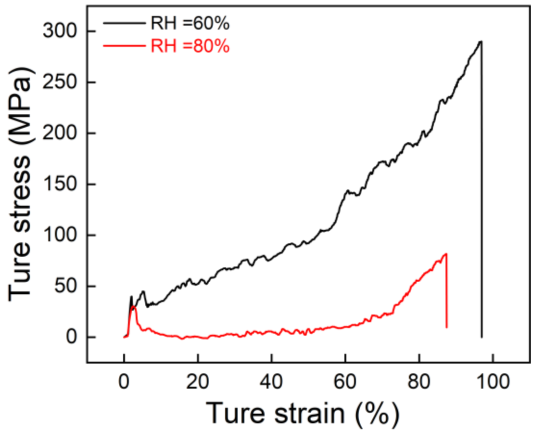

Figure S26 True stress-strain curves of hydrogel microfibers at relative humidity of 60% and 80%.

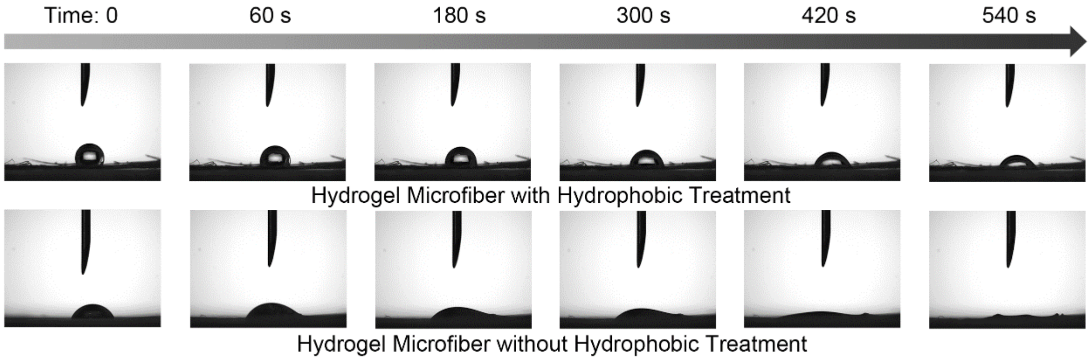

Figure S27. Time-dependent water contact angle changes of hydrogel microfiber nets with and without hydrophobic finishing agents.

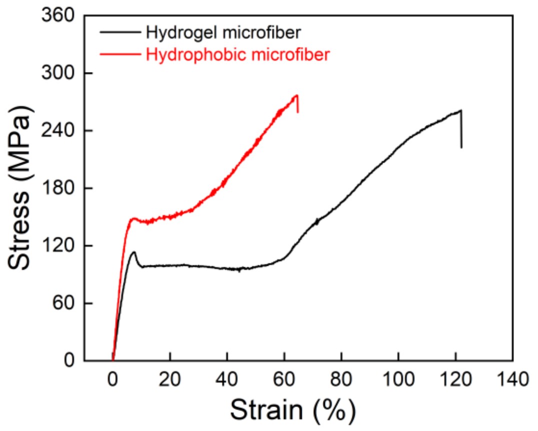

Figure S28. Stress-strain curves of hydrogel microfibers with and without hydrophobic finishing agents.

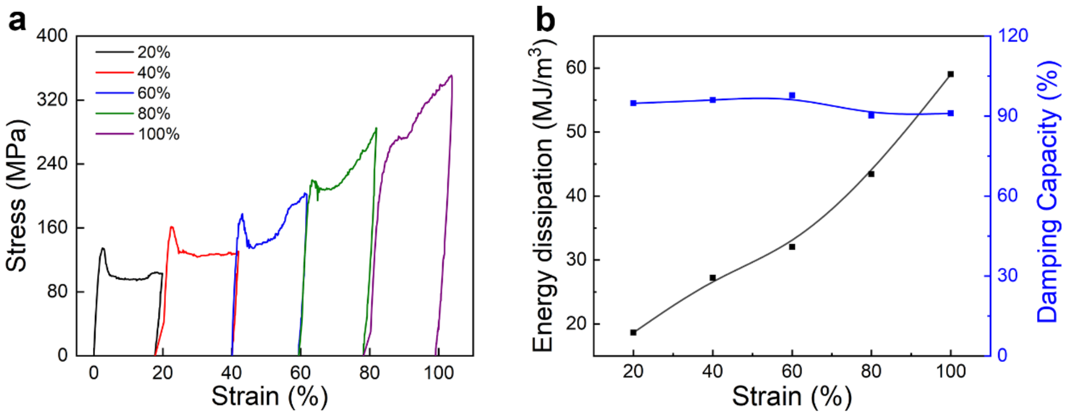

Figure S29. a) Progressive loading-unloading curves of the hydrogel fiber, b) Energy-dissipation and damping capacity of the hydrogel microfiber during progressive loading-unloading experiments.

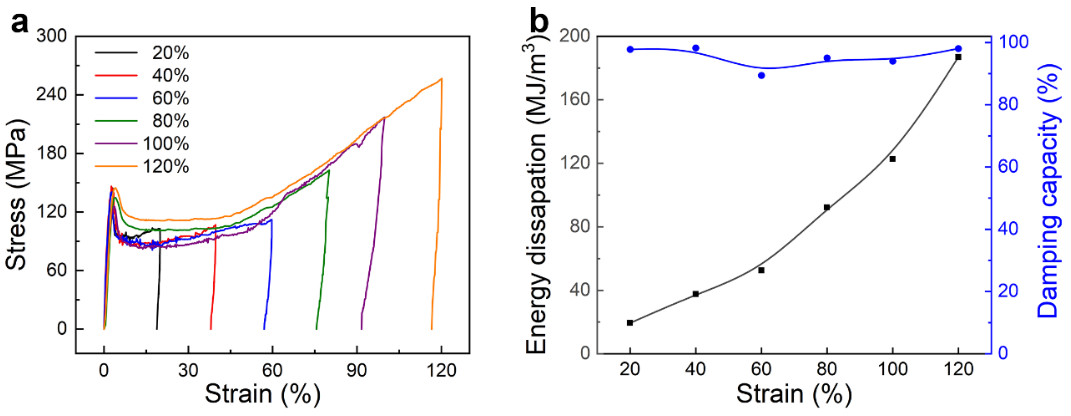

Figure S30. a) Loading-unloading curves and b) Energy-dissipation and damping capacity of hydrogel microfibers with different stretches.

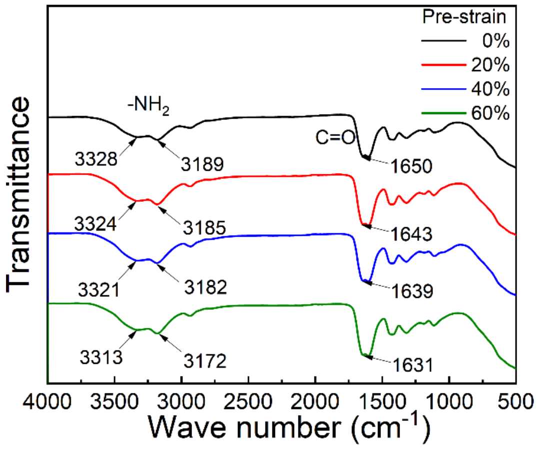

Figure S31. Fourier transform infrared spectra of hydrogel microfibers with different pre-stretch strain.[11]

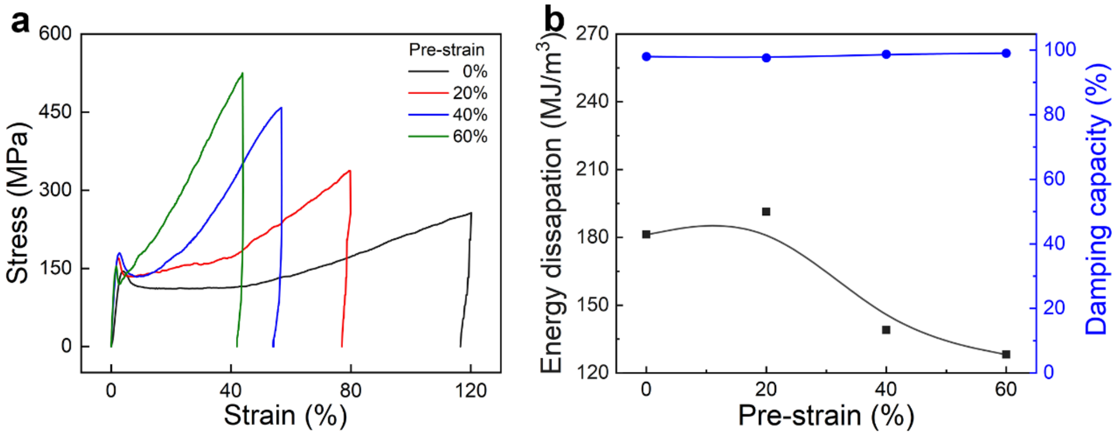

Figure S32. a) Loading-unloading curves and b) Energy-dissipation and damping capacity of hydrogel microfibers with different pre-stretch strain.

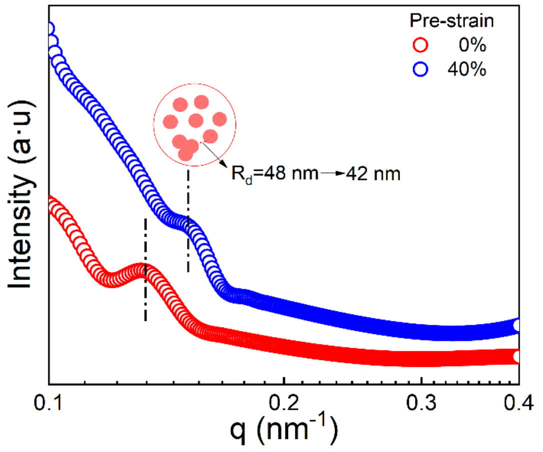

Figure S33. 1D SAXS pattern of hydrogel microfibers with different pre-stretch strain.

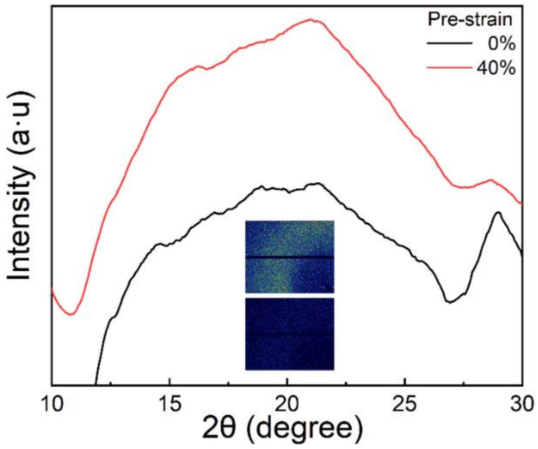

Figure S34. WAXS spectrum of hydrogel microfibers with different pre-stretch strain.

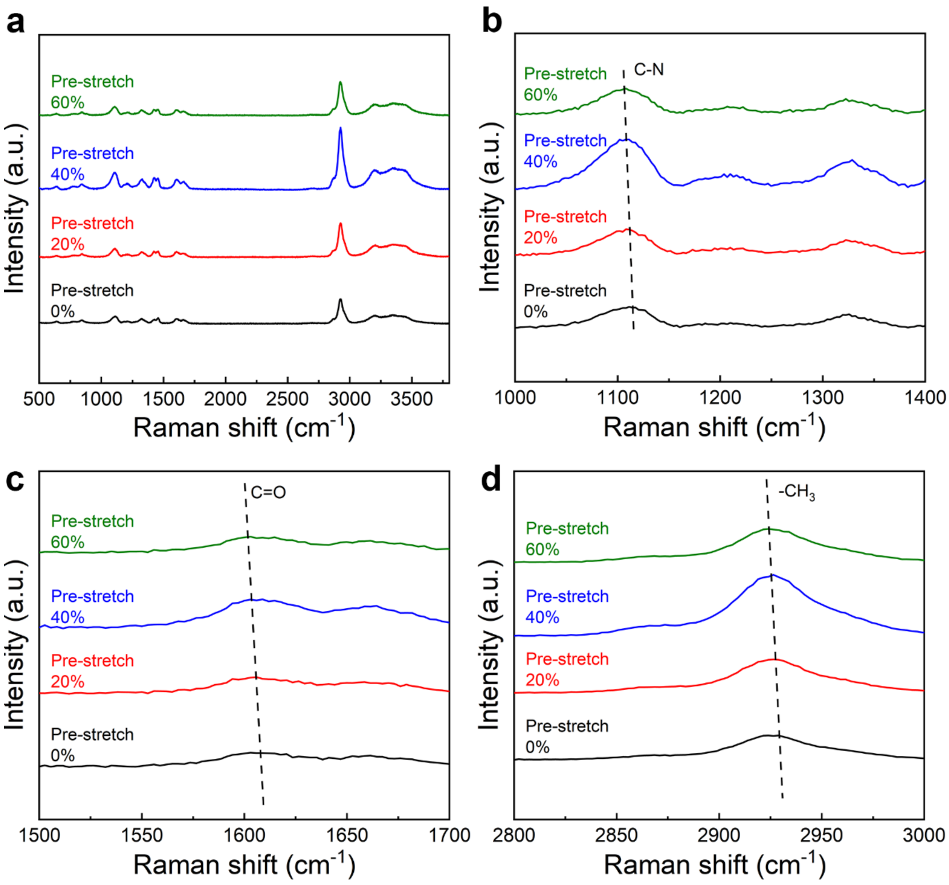

Figure S35. a) Raman spectra of hydrogel microfibers with different pre-stretch strain. b) C-N and c) C=O and d) $-CH_3$ inside hydrogel microfibers were shifted.

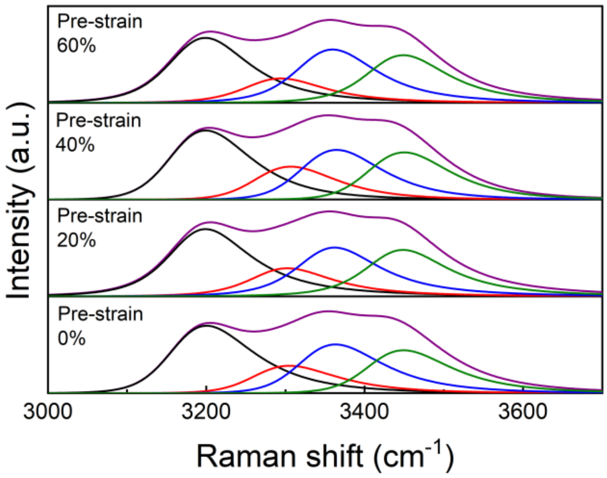

Figure S36. Raman spectra and fitting results at 3300-3700 cm^{-1} of hydrogel microfibers with different pre-stretch strain.

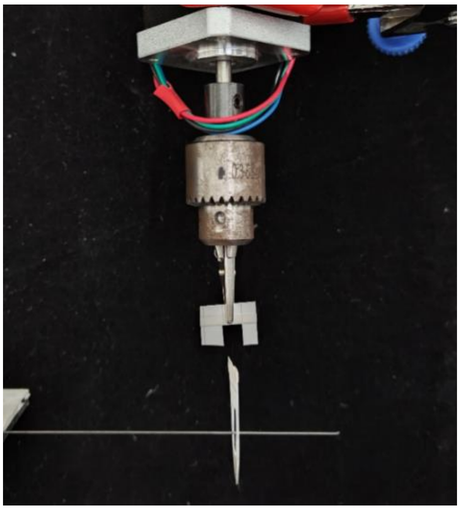

Figure S37. Twisting insertion of the hydrogel microfiber.

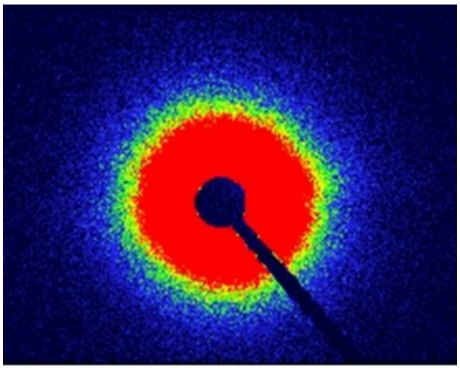

Figure S38. SAXS scattering diagram of twisted hydrogel microfibers.

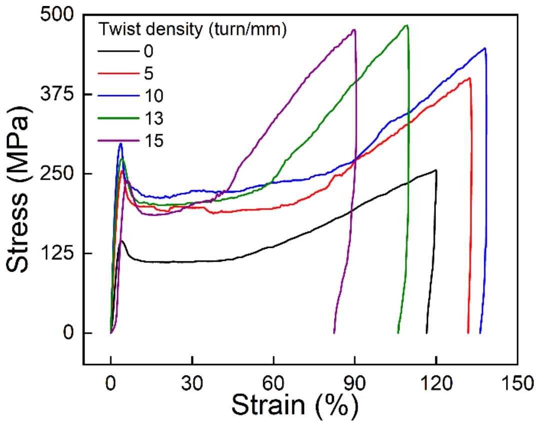

Figure S39. Loading-unloading curves of hydrogel microfibers with different twist densities.

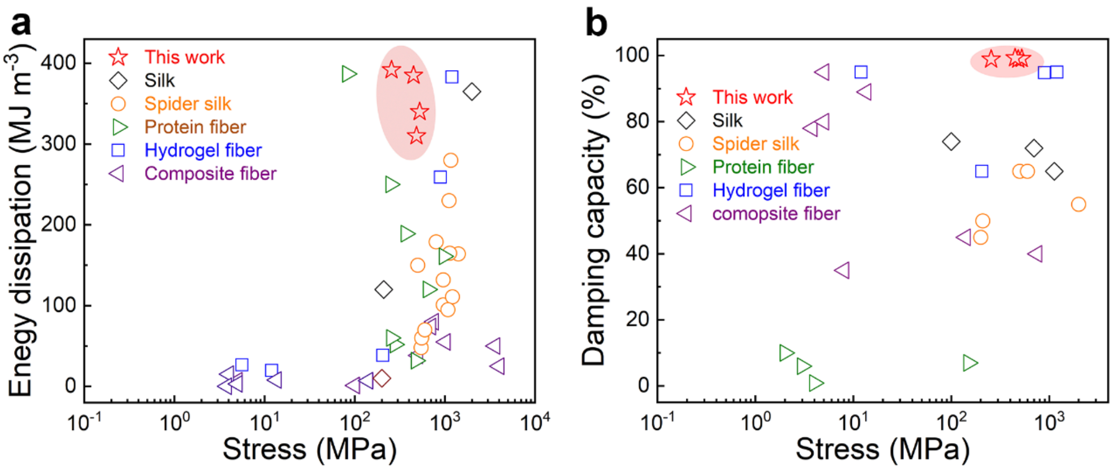

Figure S40. a) Comparison of energy-dissipation and stress of the hydrogel microfiber in this work with those of other fibrous materials. b) Comparison of damping capacity and stress of the hydrogel microfiber in this work with those of other materials. These data are presented in Supplementary Tables S1 and S2.

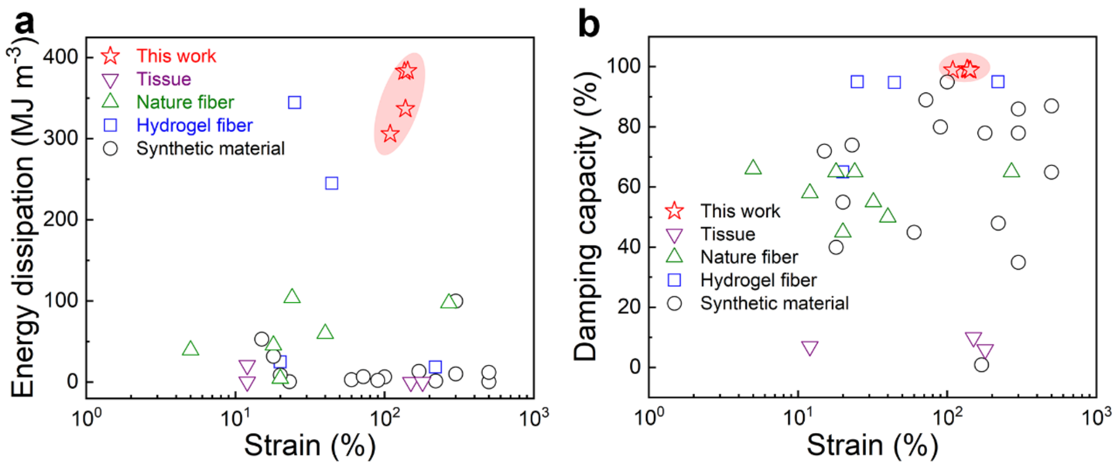

Figure S41. a) Comparison of energy-dissipation and strain of the hydrogel microfiber in this work with those of other common energy-dissipating materials. b) Comparison of damping capacity and strain of the hydrogel microfiber in this work with those of other materials. These data are presented in Supplementary Tables S1 and S2.

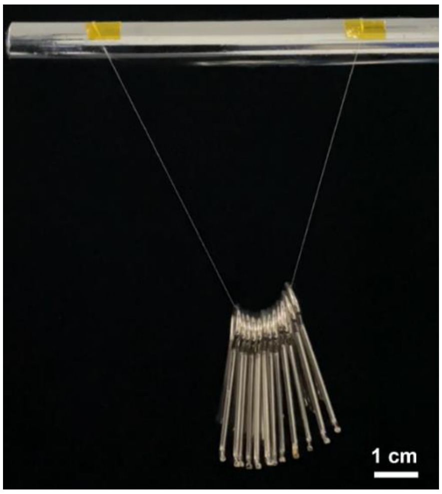

Figure S42. Hydrogel yarn contained 50-ply, 15-µm-diameter microfibers was able to carry 12 hooks weighing 1 g without breaking (Supplementary Video 2).

Supplementary Tables

Table S1. Comparison of the mechanical properties of hydrogel fibers, spider silk, and other state-of-the-art synthetic fibers in the literature.

Materials	Toughness (MJ m^{-3})	Breaking Stress (MPa)	Breaking Strain (%)	Energy Dissipation (MJ m^{-3})	Damping capacity (%)	Ref.
Hydrogel fibers	385	525	143	384	99.3	This work
Bagworm silk	365	2000	32	201	55	[12]
Regenerated silk	10	200	20	4.5	45	[13]
Amorphous silk	120	210	40	60	50	[14]
MA spider silk	160	1100	27	104	65	[15]
Viscid spider silk	150	500	270	97	65	[15]
Cocoon silk	70	600	18	35	50	[15]
C. Darwin silk	354	1652	52	N/A	N/A	[16]
Artificial spider silk	165	1130	24	104	65	[17]
SPCH fiber	38.5	205	20	25	65	[18]
PAA hydrogel fiber	259	895	44.3	245	94.8	[19]
PMAA fiber	19.8	12	219	18	95	[9]
Nanogel fiber	383	1200	25	344	95	[10]
PR hydrogel fiber	466	1600	72	N/A	N/A	[20]
Supercontractile fiber	1.0	100	23	0.8	74	[21]
Viscose fiber	74	700	12	53	72	[21]
Cellulose nanofibrils	32	478	10	N/A	N/A	[22]
Carbon fiber	25	4000	1.3	1	4	[15]
Nylon 6,6	80	750	18	32	40	[19]
Kevlar 49	50	3000	2.7	10	65	[17]

Table S2. Comparison of the mechanical properties of common natural materials and synthetic materials.

Materials	Toughness (MJ m^{-3})	Breaking Stress (MPa)	Breaking Strain (%)	Energy Dissipation (MJ m^{-3})	Damping capacity (%)	Ref.
Hydrogel fibers	385	525	143	384	99	This work
Wool	60	200	5	39.6	66	[23]
Tendon collagen	7.5	150	1.5	0.5	7	[24]
Elastin	2	1	150	0.2	10	[24]
Resilin	4	3	190	0.2	6	[24]
Cotton	36	500	12	21	58	[21]
SBR 05 Rubber	0.1	3.8	179	0.1	78	[25]
Synthetic Rubber	100	50	850	N/A	N/A	[26]
Graphene aerogel	0.3	1.2	-50	0.14	60	[27]
CNT Array	0.3	1.6	-72	1.0	64	[28]
CNT Sponge	2.4	5	-80	1.7	80	[29]
DN hydrogel	1.0	0.2	500	0.7	65	[30]
Dual-crosslinked hydrogel	14	4.5	500	12.2	87	[31]
Alginate gel	18	25.8	20	9	55	[32]
Gel-nacre	125	32	300	100	78	[33]
Nanocomposite hydrogels	3.5	0.4	220	1.7	48	[34]
Dynamic fiber	2.7	13	50	N/A	N/A	[35]
PAA-Chitosan Hydrogels	2.5	3.8	300	3.2	78	[36]
Elastomer	7.9	13.5	72	7	89	[37]
Nanocomposite Hydrogels	6.2	0.1	8000	3.5	56	[38]

Supplementary Videos

Supplementary Video 1. Continuous spinning of the spinning dope. The hydrogel fiber is continuously drawn from a spinning dope containing monomer (15 wt%) with 0.75 wt% initiator relative to the monomer by a 32-mm-diameter wheel roller at a reeling speed of 10 rpm. The distance between the spinning dope surface and the roller is 10 cm, the temperature is 20°C, and the relative humidity was 25%.

Supplementary Video 2. Loading and energy dissipation capability of hydrogel yarn with a horizontal configuration and V-shaped configuration. A total of 12 hooks with 1g of weight were placed one by one on the hydrogel yarn which were placed horizontally. The hydrogel yarn consisted of 15-cm-long, 50-ply, 15-μm-diameter hydrogel microfibers.

Supplementary Video 3. Impact force reduction of a moving object by hydrogel microfiber. A net consisted of 50-ply, 15-μm-diameter hydrogel microfibers in the vertical and horizontal directions, which received a ping-pong ball dropped from a height of 15 cm without damage.

Supplementary Video 4. Moisture-induced supercontraction of hydrogel yarn. A 5 g hook was caught by a hydrogel yarn containing 20-ply, 15-μm-diameter, 15-cm-long hydrogel microfibers. The hydrogel yarn deformed and the recovered to its original shape upon exposure to high humidity.

Reference

[1] S. Weng, *Fourier transform infrared spectrum analysis*, Chemical Industry Press, Beijing, China **2010**.

[2] S. Liu, M. Zhang, B. Huang, N. Wu, S. Ouyang, *Molecules* **2019**, *24*, 3666.

[3] Q. Hu, S. Ouyang, J. Li, Z. Cao, *J. Raman Spectrosc.* **2017**, *48*, 610.

[4] F. Zhao, X. Zhou, Y. Shi, X. Qian, M. Alexander, X. Zhao, S. Mendez, R. Yang, L. Qu, G. Yu, *Nat. Nanotechnol.* **2018**, *13*, 489.

[5] F. Zhu, L. Wang, B. Demir, M. An, Z. L. Wu, J. Yin, R. Xiao, Q. Zheng, J. Qian, *Mater. Horizons* **2020**, *7*, 3187.

[6] H. Pan, Y. Zhang, H. Shao, X. Hu, X. Li, F. Tian, J. Wang, *J. Mat. Chem. B* **2014**, *2*, 1408.

[7] L. Liu, M. Zhu, X. Xu, X. Li, Z. Ma, Z. Jiang, A. Pich, H. Wang, P. Song, *Adv. Mater.* **2021**, *33*, 2105829.

[8] P. Song, Z. Xu, M. S. Dargusch, Z. G. Chen, H. Wang, Q. Guo, *Adv. Mater.* **2017**, *29*, 1704661.

[9] Y. Shi, B. Wu, S. Sun, P. Wu, *Nat. Commun.* **2023**, *14*, 1370.

[10] W. He, D. Qian, Y. Wang, G. Zhang, Y. Cheng, X. Hu, K. Wen, M. Wang, Z. Liu, X. Zhou, M. Zhu, *Adv. Mater.* **2022**, *34*, 2201843.

[11] Z. Han, P. Wang, Y. Lu, Z. Jia, S. Qu, W. Yang, *Sci. Adv.* **2022**, *8*, eabl5066.

[12] T. Yoshioka, T. Tsubota, K. Tashiro, A. Jouraku, T. Kameda, *Nat. Commun.* **2019**, *10*, 1469.

[13] S. Ling, Z. Qin, C. Li, W. Huang, D. L. Kaplan, M. J. Buehler, *Nat. Commun.* **2017**, *8*, 1387.

[14] K. Yazawa, A. D. Malay, N. Ifuku, T. Ishii, H. Masunaga, T. Hikima, K. Numata, *Biomacromolecules* **2018**, *19*, 2227.

[15] J. M. Gosline, P. A. Guerette, C. S. Ortlepp, K. N. Savage, *J. Exp. Biol.* **1999**, *202*, 3295.

[16] I. Agnarsson, M. Kuntner, T. A. Blackledge, *PLoS One* **2010**, *5*, e11234.

[17] A. Rising, J. Johansson, *Nat. Chem. Biol.* **2015**, *11*, 309.

[18] Y. Wu, D. U. Shah, C. Liu, Z. Yu, J. Liu, X. Ren, M. J. Rowland, C. Abell, M. H. Ramage, O. A. Scherman, *Proc. Natl. Acad. Sci. U. S. A.* **2017**, *114*, 8163.

[19] Y. Dou, Z. Wang, W. He, T. Jia, Z. Liu, P. Sun, K. Wen, E. Gao, X. Zhou, X. Hu, J. Li, S. Fang, D. Qian, Z. Liu, *Nat. Commun.* **2019**, *10*, 5293.

[20] J. Sun, W. Guo, G. Mei, S. Wang, K. Wen, M. Wang, D. Feng, D. Qian, M. Zhu, X. Zhou, Z. Liu, *Adv. Mater.* **2023**, *35*, 2212112.

[21] Y. Wu, D. U. Shah, B. Wang, J. Liu, X. Ren, M. H. Ramage, O. A. Scherman, *Adv. Mater.* **2018**, *30*, 1707169.

[22] P. Mohammadi, A. S. Aranko, C. P. Landowski, O. Ikkala, K. Jaudzems, W. Wagermaier, M. B. Linder, *Sci. Adv.* **2019**, *5*, eaaw2541.

[23] J. Wu, L. H. Cai, D. A. Weitz, *Adv. Mater.* **2017**, *29*, 1702616.

[24] Y. Yu, Y. He, Z. Mu, Y. Zhao, K. Kong, Z. Liu, R. Tang, *Adv. Funct. Mater.* **2019**, *30*, 1908556.

[25] C. M. Roland, *J. Rheol.* **1989**, *33*, 659.

[26] M. Elices, G. R. Plaza, J. Pérez Rigueiro, G. V. Guinea, *J. Mech. Behav. Biomed. Mater.* **2011**, *4*, 658.

[27] C. Zhu, T. Y. J. Han, E. B. Duoss, A. M. Golobic, J. D. Kuntz, C. M. Spadaccini, M. A. Worsley, *Nat. Commun.* **2015**, *6*, 6962.

[28] Z. Zeng, X. Gui, Q. Gan, Z. Lin, Y. Zhu, W. Zhang, R. Xiang, A. Cao, Z. Tang, *Nanoscale* **2014**, *6*, 1748.

[29] X. Gui, Z. Zeng, Y. Zhu, H. Li, Z. Lin, Q. Gan, R. Xiang, A. Cao, Z. Tang, *Adv. Mater.* **2013**, *26*, 1248.

[30] Q. Chen, L. Zhu, C. Zhao, Q. Wang, J. Zheng, *Adv. Mater.* **2013**, *25*, 4171.

[31] P. Lin, S. Ma, X. Wang, F. Zhou, *Adv. Mater.* **2015**, *27*, 2054.

[32] M. T. I. Mredha, Y. Z. Guo, T. Nonoyama, T. Nakajima, T. Kurokawa, J. P. Gong, *Adv. Mater.* **2018**, *30*, 1704937.

[33] X. Zhao, M. Wang, Y. Chen, Z. Chen, T. Suo, W. Qian, J. Hu, X. Song, W.-N. Mei, R. Sabirianov, L. Tan, *ACS Appl. Mater. Interfaces* **2019**, *11*, 19421.

[34] M. Zhong, F. K. Shi, Y. T. Liu, X. Y. Liu, X. M. Xie, *Chin. Chem. Lett.* **2016**,

27, 312.

[35] C. K. Chu, A. J. Joseph, M. D. Limjoco, J. Yang, S. Bose, L. S. Thapa, R. Langer, D. G. Anderson, *J. Am. Chem. Soc.* **2020**, *142*, 19715.

[36] Y. Yang, X. Wang, F. Yang, H. Shen, D. Wu, *Adv. Mater.* **2016**, *28*, 7178.

[37] E. Filippidi, T. R. Cristiani, C. D. Eisenbach, J. H. Waite, J. N. Israelachvili, B. K. Ahn, M. T. Valentine, *Science* **2017**, *358*, 502.

[38] G. Gao, G. Du, Y. Sun, J. Fu, *ACS Appl. Mater. Interfaces* **2015**, *7*, 5029.
